# Facile Amidation of Non-Protected Hydroxycinnamic Acids for the Synthesis of Natural Phenol Amides

**DOI:** 10.3390/molecules27072203

**Published:** 2022-03-28

**Authors:** Annemiek van Zadelhoff, Jean-Paul Vincken, Wouter J. C. de Bruijn

**Affiliations:** Laboratory of Food Chemistry, Wageningen University, 6708 WG Wageningen, The Netherlands; annemiek.vanzadelhoff@wur.nl (A.v.Z.); jean-paul.vincken@wur.nl (J.-P.V.)

**Keywords:** hydroxycinnamic acid amides (HCAAs), phenyl amides, avenanthramides, amidation

## Abstract

Phenol amides are bioactive compounds naturally present in many plants. This class of compounds is known for antioxidant, anti-inflammatory, and anticancer activities. To better understand the reactivity and structure–bioactivity relationships of phenol amides, a large set of structurally diverse pure compounds are needed, however purification from plants is inefficient and laborious. Existing syntheses require multiple steps, including protection of functional groups and are generally overly complicated and only suitable for specific combinations of hydroxycinnamic acid and amine. Thus, to facilitate further studies on these promising compounds, we aimed to develop a facile general synthetic route to obtain phenol amides with a wide structural diversity. The result is a protocol for straightforward one-pot synthesis of phenol amides at room temperature within 25 h using equimolar amounts of *N*,*N*′-dicyclohexylcarbodiimide (DCC), amine, hydroxycinnamic acid, and sodium bicarbonate in aqueous acetone. Eight structurally diverse phenol amides were synthesized and fully chemically characterized. The facile synthetic route described in this work is suitable for a wide variety of biologically relevant phenol amides, consisting of different hydroxycinnamic acid subunits (coumaric acid, ferulic acid, and sinapic acid) and amine subunits (agmatine, anthranilic acid, putrescine, serotonin, tyramine, and tryptamine) with yields ranging between 14% and 24%.

## 1. Introduction

Phenol amides, also referred to as hydroxycinnamic acid amides, consist of a phenolic subunit (i.e., a hydroxycinnamic acid) linked to an amine subunit via an amide bond [1]. These compounds are commonly found as the main phenolic compounds present in the reproductive organs and seeds of many different plant species and are known to accumulate in high concentrations in wounded plant tissue [1,2]. Phenol amides are compounds of interest due to their bioactivity, as compounds within this class are known to possess antioxidant, anti-inflammatory, and anticancer activity [3].

**Figure 1:**
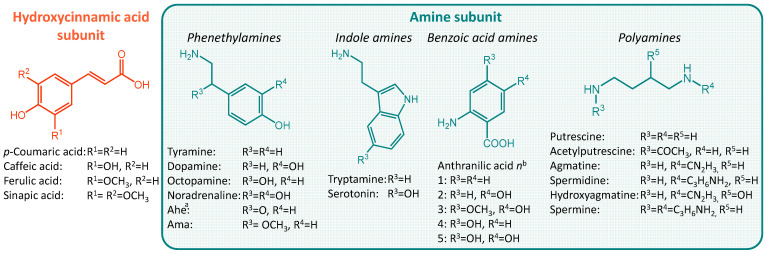
Overview of hydroxycinnamic acid and amine subunits present in naturally occurring phenol amides [4,5]. ^a^ Ahe, 2-amino-1-(4-hydroxyphenyl)ethanone; Ama, α-(aminomethyl)-4-methoxybenzyl alcohol ^b^ The n in Antn is replaced by the number of the corresponding acid.

They can consist of different hydroxycinnamic acid subunits, most commonly *p*-coumaric acid, ferulic acid, sinapic acid, and caffeic acid, and different amine subunits, most commonly tyramine, agmatine, anthranilic acid, tryptamine, and putrescine. Therefore, phenol amides with a very wide variety of structural features can be found in nature (Figure 1) [1,3]. Abbreviations for phenol amides used in this study are in line with abbreviations used to indicate the phenol amide composition in lignanamides [4].

Despite the fact that their bioactivity has been well established, it has recently been emphasized in three different reviews that further investigation of their possible beneficial effects on human health is needed [1,3,5].

Purification of phenol amides from plants is time consuming and requires a large amount of plant material and solvents. Therefore, organic synthesis of individual phenol amides is essential to aid future studies on their biofunctionality and to establish structure–activity relationships. One of the main problems with currently used amidation protocols is that most of these protocols have not been tested for the diverse combinations of phenol and amine subunits that are commonly present in plants. The more complex plant phenol (e.g., ferulic acid) and amine (e.g., tyramine) subunits are generally less water-soluble; therefore, commonly used aqueous amidation protocols are not suitable for the synthesis of the full diversity of phenol amides from plants. Furthermore, existing amidation protocols in organic solvents are not ideal alternatives either, as these protocols often use hazardous and unsustainable solvents such as dimethylformamide, *N*-methylpyrrolidon, or dichloromethane. The use of these solvents is currently considered one of the main issues in amidation reactions, emphasizing the importance of the use of alternative, greener solvents [6].

Moreover, existing protocols are multi-step reactions, which require protection of functional groups, use of elevated temperatures, use of metal catalysts, or total reaction times of at least two days [7,8,9,10]. Many general amidation protocols have been reported in literature. For an overview of existing protocols, we refer the reader to three recent reviews [11,12,13]. Furthermore, Fattahi et al. [14] provided a comprehensive overview of synthetic methods for aromatic amides.

In this study, the aim was to develop a general, facile protocol for the synthesis of phenol amides from a variety of hydroxycinnamic acid and amine subunits using mild conditions and non-hazardous chemicals. The starting compounds used, hydroxycinnamic acids and amines, are affordable and commercially available. The amidation protocol tested in this work is inspired by the protocols of Stöeckigt and Zenk (1975) [15], Negrel et al. (1984) [9], and Muroi et al. (2009) [16]. Adaptations were made based on the method of Alamgir et al. [17] for the synthesis of *p*-coumaroylputrescine and feruloylputrescine using *N*,*N*′-dicyclohexylcarbodiimide (DCC) and the method of Fattahi et al. [14] for the synthesis of small and more polar amides using *N*,*N*′-diisopropylcarbodiimide (DIC). Reaction conditions were optimized to establish a one-pot synthetic route suitable for a wide variety of biologically relevant phenol amides.

## 2. Results and Discussion

Multiple amidation protocols from literature were tested. In an attempt to avoid the use of organic solvents, a green protocol using water-soluble *N*,*N*′-diisopropylcarbodiimide (DIC) instead of water insoluble *N*,*N*′-dicyclohexylcarbodiimide (DCC) was tested. However, when using this protocol from Fattahi et al. (2018) [14], the reaction of the hydroxycinnamic acid with *N*,*N*′-diisopropylcarbodiimide resulted in the formation of a large insoluble lump. A tenfold reduction in the concentrations of all reactants did not alleviate these solubility issues. In the same paper, Fattahi et al. [14] presented an overview of amidation protocols. These protocols and other protocols described in literature have not been tested for the compounds of interest for our study, use elevated temperatures, use metals or unsustainable solvents, multi-step protocols, or expensive reagents [14,17,18,19,20,21]. As we aimed to develop a facile one-pot protocol, these protocols were not considered to be suitable starting points and were, therefore, not tested.

Based on our experiments using water and DIC, water is not a suitable solvent. Therefore, a protocol based on the use of DCC in organic solvents was tested [9,15,16]. Different protocols were tested for synthesis of feruloyltyramine (FerTrm) and feruloyltryptamine (FerTry). Tyramine and tryptamine were used as these two compounds are among the most studied amine subunits in natural phenol amides and represent two common types of amine subunits, the phenol and indole type, respectively [5]. For validation of the final protocol, the protocol was tested for ferulic acid in combination with six of the most common amines, which included representatives from each of the four different types of amines. Specifically for the alkyl amines both agmatine and putrescine were tested, as agmatine can form phenol amides with one amide bond, whereas putrescine is a diamine capable of forming amide bonds with two hydroxycinnamic acid subunits [3]. Besides different amines, three hydroxycinnamic acids were tested in combination with agmatine to synthesize three types of phenol amides naturally occurring in barley [22]. To optimize our synthesis, three different protocols were tested in which variations in different conditions were assessed simultaneously for each protocol. More detailed descriptions of the conditions used during the optimization of our synthesis, and the conversions obtained are given in the supplementary information (Table S1). Conversion was determined using LC-MS analysis of the crude reaction mixture. Purification and determination of isolated yield was only performed for the products of the final synthesis protocol.

The first protocol tested was a two-step reaction, starting with a 24-h incubation of equimolar amounts of hydroxycinnamic acid, *N*-hydroxysuccinimide, and DCC in ethyl acetate, as described by Stöeckigt and Zenk (1975) [15]. After filtration to remove the insoluble dicyclohexyludea (DCU) formed and removal of ethyl acetate under reduced pressure, the remaining ester was dissolved in acetone and incubated for 24 h with an equimolar amount of amine and 2.5 molar equivalents of aqueous NaHCO_3_ (protocol 1, condition 1), based on the protocols described by Negrel et al. (1984) [9] and Muroi et al. (2009) [16]. Condition 1 resulted in conversions of 12.3% and 3.4% for FerTrm and FerTry, respectively. Extending the incubation time to 48 h (protocol 1, condition 2), did not result in a sufficient increase in conversion.

To simplify and optimize the synthesis, a one-pot approach was tested as the second protocol. This one-pot approach was optimized by varying different conditions as described below. First, simultaneous addition of hydroxycinnamic acid, amine, DCC, and *N*-hydroxysuccinimide in acetone to 2.5 molar equivalents of aqueous NaHCO_3_ was tested. After 24 h of incubation (protocol 2, condition 3), both FerTrm and FerTry were synthesized with a 1.1% conversion. Extending the incubation to 48 h (protocol 2, condition 4) did not notably increase the conversions. Subsequently, this one-pot approach was tested in absence of *N*-hydroxysuccinimide (protocol 2, condition 5), with 4 molar equivalents of NaHCO_3_ (protocol 2, condition 6), by using NaOH instead of NaHCO_3_ (protocol 2, condition 7), and by adding 2 molar equivalents of amine (protocol 2, condition 8). None of these adaptations resulted in improvement of the conversion obtained with the original conditions of the second protocol (Table S1, supplementary information). In the different one-pot reactions tested with protocol 2, between 100% and 65% of the hydroxycinnamic acid remained unreacted and various by-products were formed. The reaction in which 4 molar equivalents of NaHCO_3_ were added (protocol 2, condition 6) resulted in a ferulic acid conversion of 89%, however phenol amide formation at this condition was still very limited due to extensive by-product formation.

Therefore, to improve the activation of the hydroxycinnamic acid with DCC, protocol three consisted of a pre-incubation of equimolar amounts of hydroxycinnamic acid and DCC (without addition of *N*-hydroxysuccinimide) in acetone for one hour, before addition of equimolar amounts of amine. This protocol was tested with (protocol 3, condition 9) and without (protocol 3, condition 10) equimolar amounts of aqueous NaHCO_3_. After incubation for 24 h the protocol with equimolar amounts of NaHCO_3_ resulted in conversions of 43.8% and 10.0% for FerTrm and FerTry, respectively. Overall, this reaction (protocol 3, condition 10) resulted in increased conversions, shorter reaction time, fewer steps, and the use of fewer reagents, compared to protocol 1 and 2.

Based on the aforementioned optimization, the final protocol, which resulted in the highest conversion for FerTrm and FerTry, was a one-pot reaction with a DCC and hydroxycinnamic acid pre-incubation using equimolar amounts of hydroxycinnamic acid, amine, DCC, and sodium bicarbonate. This protocol was then applied on a larger scale to three types of hydroxycinnamic acids (coumaric acid, ferulic acid, and sinapic acid) and six types of amines (agmatine, anthranilic acid, putrescine, serotonin, tryptamine, and tyramine). The products obtained were purified and characterized. The structure, purity, conversion, and yields of the obtained compounds are shown in Table 1. All characterization data of the obtained compounds is provided in the Supplementary information (Table S2 and Figures S1–S19).

The method used as the starting point for the development of protocol 1 was reported to result in a yield of 22.9% [16] for coumaroylagmatine (CouAgm), however the highest yield obtained using this protocol in this study was 12.3% for feruloylagmatine (FerAgm) and FerTrm. A similar protocol using DIC in water was reported to result in yields between 87% and 96% [14]; however, due to solubility issues, this protocol was not suitable for synthesis of the phenol amides of interest.

To conclude, the simplified one-pot protocol presented in this paper has numerous advantages compared to previously reported protocols, namely, it does not require temperature control, can be performed using water and acetone as greener solvents, uses shorter reaction times, requires fewer reactants, and does not include any intermediate filtration, evaporation, or purification steps. Even though the yields obtained are lower compared to some other amidation protocols, our yields are still reasonable. Overall, the presented protocol allows for facile synthesis of phenol amides.

## 3. Materials and Methods

### 3.1. General Experimental Information

All reagents and solvents used were purchased from commercial sources and used without further purification. Column chromatography was performed on a Pure C-850 FlashPrep system (Büchi Flawil, Switzerland) operated in flash mode, to purify the products. The eluents used were water with 1% (*v*/*v*) FA and ACN with 1% (*v*/*v*) FA. Separation was performed on a 12 g FlashPure ID C18 column (Büchi). For analysis of the phenol amides the samples were separated on a Thermo Vanquish UHPLC system (Thermo Scientific, San Jose, CA) equipped with a pump, degasser, autosampler, and PDA detector, using a Waters Acquity BEH C18 column (150 mm × 2.1 mm i.d., 1.7 μm particle size) with a VanGuard guard column of the same material (5 mm × 2.1 mm i.d., 1.7 μm particle size) (Waters, Milford, MA, USA). Mass spectrometric data were acquired using a LTQ Velos Pro linear ion trap mass spectrometer (Thermo Scientific) equipped with a heated ESI probe coupled in-line to the RP-UHPLC system. High-resolution mass spectra were recorded on a Q Exactive Focus hybrid quadrupole-Orbitrap mass spectrometer (Thermo Scientific), equipped with a heated ESI probe, coupled to a Vanquish RP-UHPLC system. NMR spectra were recorded at a probe temperature of 300 K on a Bruker Avance-III-600 spectrometer (Bruker, Billerica, MA, USA) located at the MAGNEtic resonance research FacilitY of Wageningen University. For all compounds, 1D ^1^H and ^13^C and 2D HMBC and HMQC spectra were acquired. UV–Vis spectra were recorded using a Genesis 150 UV/Vis spectrophotometer (Thermo Scientific). Melting points were determined using a Q200 differential scanning calorimeter (DSC) (TA Instruments, New Castle, DE, USA). Attenuated total reflectance Fourier transform infrared (ATR-FTIR) spectra were recorded using an Invenio-S (Bruker, Billeria, MA, USA) equipped with a BioATRcell II (Harricks, Pleasantville, NY, USA). More detailed information on the methods used is provided in the supplementary information.

### 3.2. General Procedure for the Synthesis of Compounds ***1***–***8***

All protocols used for optimization of the synthesis are given in the supplementary information (Table S1). This section focusses on the optimized final protocol.

To a 0.072 M solution of *N*,*N*′-dicyclohexylcarbodiimide (DCC) in acetone, an equimolar amount of hydroxycinnamic acid was added. The solution was stirred for 1 h at room temperature in the dark. Photoisomerization has been reported for several phenol amides [23,24], therefore exposure to light was limited as much as possible during all syntheses. After 1 h an equimolar amount of the desired amine was added, followed by addition of a 0.072 M sodium bicarbonate solution in a volume equal to the starting volume of acetone. The mixture was stirred for 24 h at room temperature and in the dark. After 24 h the reaction was stopped by adding an equimolar amount of acetic acid. Insoluble *N*,*N*′-dicyclohexylurea (DCU) formed was removed by filtration using a cellulose filter. A sample of 200 μL was taken and centrifuged (10,000× *g*, 5 min) prior to analysis by UHPLC-PDA-ESI-IT-MS. For the remaining reaction mixture, acetone was removed under reduced pressure at 40 °C after which the remaining water phase was washed three times with ethyl acetate. For further purification by flash chromatography, the ethyl acetate fraction was used for phenol amides with anthranilate, serotonin, tyramine, and tryptamine subunits. For phenol amides with agmatine or putrescine subunits, the water fraction was used. To the ethyl acetate samples 5 mL water was added prior to concentration. All samples were concentrated under reduced pressure at 60 °C to remove the ethyl acetate before purification by flash chromatography.

**Coumaroylagmatine (1)**. White powder. M.p. nd. UV-Vis (water, λ_max_, nm): 192, 297. IR (water, ν_max_, cm^–1^): 1654, 1587, 1516, 1229, 1128, 1102. ^1^H NMR (600 MHz, CD_3_OD, δ in ppm, *J* in Hz): δ 1.66 (H, m, H-12/H-13), 3.32 (2H, m, H-11), 3.35 (2H, m, H-14), 6.49 (1H, d, *J* = 15.60 Hz, H-8), 6.81 (2H, 6, *J* = 8.70 Hz, H-3/H-5), 7.43 (2H, d, *J* = 8.55 Hz, H-2/H-6), 7.47 (1H, d, *J* = 16.05 Hz, H-7). ^13^C NMR (150 MHz, CD_3_OD, δ in ppm): δ 168.1 (C-9), 159.1 (C-4), 157.3 (C-16), 140.3 (C-7), 129.2 (C-2/C-6), 126.3 (C-1), 114.1 (C-8), 115.3 (C-3/C-5), 40.7 (C-14), 38.5 (C-11), 26.2 (C-13), 25.7 (C-12). ESI-Orbitrap-MS 277.16559 [M + H]^+^ (−1.13 ∆ppm), C_14_H_20_N_4_O_2_.

**Sinapoylagmatine (2)**. White powder. M.p. 62 °C. UV (water λ_max_, nm): 193, 234, 315. IR (water, ν_max_, cm^–1^): 2943, 1675, 1565, 1514, 1459, 1424, 1330, 1209, 1099, 1066, 973. ^1^H NMR (600 MHz, CD_3_OD, δ in ppm, *J* in Hz): δ 1.67 (4H, m, H-12/H-13), 3.22 (2H, m, H-11), 3.36 (2H, m, H-14), 3.90 (6H, s, OCH_3_), 6.54 (1H, d, *J* = 15.19 Hz, H-8), 6.89 (2H, s, H-2/H-6), 7.45 (1H, d, *J* = 15.65 Hz, H-7). ^13^C NMR (150 MHz, CD_3_OD, δ in ppm): δ 168.1 (C-9), 157.4 (C-16), 148.2 (C-3/C-5), 140.9 (C-7), 137.6 (C-4), 126.0 (C-1), 118.0 (C-8), 105.2 (C-2/C-6), 55.5 (C-1′), 40.7 (C-14), 38.5 (C-11), 26.4 (C-13), 25.8 (C-12). ESI-Orbitrap-MS 337.18701 [M + H]^+^ (−0.06 ∆ppm), C_16_H_24_N_4_O_3_.

**Feruloylagmatine (3)**. Yellow powder. M.p. nd. UV (MeOH, λ_max_, nm): 199, 322. IR (water, ν_max_, cm^–1^): 3508, 2945, 1653, 1588, 1517, 1275, 1098. ^1^H NMR (600 MHz, CD_3_OD, δ in ppm, *J* in Hz): δ 1.65 (2H, m, H-13), 1.68 (2H, m, H-12), 3.24 (2H, t, *J* = 7.43 Hz, H-14), 3.37 (2H, d, *J* = 6.34 Hz, H-11), 3.91 (3H, s, OCH_3_), 6.45 (1H, d, *J* = 15.69 Hz, H-8), 6.82 (1H, d, *J* = 8.17 Hz, H-5), 7.05 (1H, dd, *J* = 8.07 Hz, 1.96 Hz, H-6), 7.14 (1H, d, *J* = 1.80 Hz, H-2), 7.47 (1H, d, *J* = 15.48 Hz, H-7). ^13^C NMR (150 MHz, CD_3_OD, δ in ppm): δ 168.1 (C-9), 157.4 (C-16), 148.8 (C-4), 148.1 (C-3), 140.9 (C-7), 126.9 (C-1), 121.9 (C-6), 117.3 (C-8), 115.3 (C-5), 110.4 (C-2), 55.0 (C-1′), 40.5 (C-14), 38.2 (C-11), 26.5 (C-13), 25.5 (C-12). ESI-Orbitrap-MS 307.17630 [M + H]^+^ (−0.55 ∆ppm), C_15_H_22_N_4_O_3_.

**Feruloyl anthranilate (4)**. Light yellow powder. M.p. 114 or 190 °C. UV (MeOH, λ_max_, nm): 218, 325. IR (water, ν_max_, cm^–1^): 3523, 2607, 1654, 1583, 1512, 1429, 1267, 1024, 986. ^1^H NMR (600 MHz, CD_3_OD, δ in ppm, *J* in Hz): δ 3.94 (3H, s, OCH_3_), 6.61 (1H, d, *J* = 15.43 Hz, H-8), 6.84 (1H, d, *J* = 8.21 Hz, H-5), 7.13 (1H, dd, *J* = 8.20 Hz, 1.96 Hz, H-6), 7.15 (1H, d, *J* = 7.87 Hz, H-10), 7.26 (1H, d, *J* = 1.96 Hz, H-2), 7.54 (1H, td, *J* = 15.77 Hz, 7.96 Hz, 1.57 Hz, H-14), 7.61 (1H, d, *J* = 15.56 Hz, H-7) 8.13 (1H, dd, *J* = 7.99 Hz, 1.42 Hz, H-13), 8.69 (1H, dd, *J* = 8.55 Hz, 0.82 Hz, H-16). ^13^C NMR (150 MHz, CD_3_OD, δ in ppm): δ 171.3 (C-17), 165.9 (C-9), 149.1 (C-4), 148.2 (C-3), 142.5 (C-7), 141.2 (C-12), 133.0 (C-14), 131.4 (C-13), 126.7 (C-1), 122.7 (C-6), 119.9 (C-16), 118.5 (C-8), 115.3 (C-5), 110.2 (C-2), 55.3 (C-1′). ESI-Orbitrap-MS 312.08768 [M − H]^−^ (−0.22 ∆ppm), C_17_H_15_NO_5_.

**Feruloylputrescine (5)**. Light yellow/beige powder. M.p. 71 °C. UV (MeOH, λ_max_, nm): 199, 233, 315. IR (water, ν_max_, cm^–1^): 2950, 2512, 1652, 1581, 1517, 1275, 1016. ^1^H NMR (600 MHz, CD_3_OD, δ in ppm, *J* in Hz): δ 1.67 (2H, m, H-13), 1.72 (2H, H-12), 2.98 (2H, t, *J* = 8.90 Hz, H-14), 3.36 (2H, d, *J* = 8.00 Hz, 5.25 Hz, H-11), 3.91 (3H, s, OCH_3_), 6.44 (1H, d, *J* = 15.61 Hz, H-8), 6.82 (1H, d, *J* = 8.16 Hz, H-5), 7.05 (1H, dd, *J* = 8.16 Hz, 1.96 Hz, H-6), 7.14 (1H, d, *J* = 1.92 Hz, H-2), 7.48 (1H, d, *J* = 15.77 Hz, H-7). ^13^C NMR (150 MHz, CD_3_OD, δ in ppm): δ 168.2 (C-9), 148.6 (C-4), 148.0 (C-3), 141.1 (C-7), 126.7 (C-1), 121.8 (C-6), 117.1 (C-8), 115.3 (C-5), 110.2 (C-2), 55.1 (C-1′), 38.9 (C-14), 38.2 (C-11), 26.3 (C-13), 24.5 (C-12). ESI-Orbitrap-MS 265.15442 [M + H]^+^ (−0.94 ∆ppm), C_14_H_20_N_2_O_3_.

**Feruloylserotonin (6)**. Beige powder. M.p. 106 °C. UV (MeOH λ_max_, nm): 206, 314. IR (water, ν_max_, cm^–1^): 2921, 2850, 2521, 1651, 1580, 1512, 1458, 1267, 1016. ^1^H NMR (600 MHz, CD_3_OD, δ in ppm, *J* in Hz): δ 2.95 (2H, t, *J* = 8.45 Hz, H-12), 3.60 (2H, t, *J* = 8.37 Hz, 7.13 Hz, H-11), 3.90 (3H, s, OCH_3_), 6.44 (1H, d, *J* = 15.65 Hz, H-8), 6.68 (1H, dd, *J* = 8.60 Hz, 2.32 Hz, H-20), 6.81 (1H, d, *J* = 8.18 Hz, H-5), 6.98 (1H, d, *J* = 2.22 Hz, H-18), 7.04 (1H, d, *J* = 2.12 Hz, H-6), 7.05 (1H, s, H-14), 7.14 (1H, d, *J* = 1.82 Hz, H-2), 7.18 (1H, d, *J* = 8.71 Hz, H-21), 7.46 (1H, d, *J* = 15.68 Hz, H-7). ^13^C NMR (150 MHz, CD_3_OD, δ in ppm): δ 168 (C-9), 149.9 (C-19), 148.7 (C-4), 147.9 (C-3), 140.8 (C-7), 131.8 (C-16), 128.3 (C-17), 127.0 (C-1), 123.0 (C-14), 122.0 (C-6), 117.5 (C-8), 115.1 (C-5), 111.3 (C-21), 110.9 (C-20), 110.2 (C-2), 102.1 (C-18), 55.1 (C-1′), 40.0 (C-11), 25.1 (C-12). ESI-Orbitrap-MS 353.14938 [M + H]^+^ (−0.57 ∆ppm), C_20_H_20_N_2_O_4_.

**Feruloyltyramine (7)**. Light yellow/beige powder. M.p. 100 °C. UV (MeOH, λ_max_, nm): 202, 320. IR (water, ν_max_, cm^–1^): 3354, 2923, 2850, 2488, 1646, 1570, 1344, 1251, 1121, 1035. ^1^H NMR (600 MHz, CD_3_OD, δ in ppm, *J* in Hz): δ 2.77 (2H, t, *J* = 8.46 Hz, H-12), 3.48 (2H, t, *J* = 8.08 Hz, 7.14 Hz, H-11), 3.90 (3H, s, OCH_3_), 6.42 (1H, d, *J* = 15.47 Hz, H-8), 6.74 (2H, d, *J* = 8.46 Hz, H-124/H-18), 6.81 (1H, d, *J* = 8.01 Hz, H-5), 7.04 (1H, dd, *J* = 8.20 Hz, 1.86 Hz, H-6), 7.07 (2H, d, *J* = 8.39 Hz, H-15/H-17), 7.14 (1H, d, *J* = 1.96 Hz, H-2), 7.45 (1H, d, *J* = 15.67 Hz, H-7). ^13^C NMR (150 MHz, CD_3_OD, δ in ppm): δ 167.9 (C-9), 155.6 (C-16), 148.6 (C-4), 147.8 (C-3), 140.7 (C-7), 130.0 (C-13), 129.5 (C-15/C-17), 126.9 (C-1), 121.9 (C-6), 117.3 (C-8), 115.2 (C-5), 114.9 (C-14/C-18), 110.1 (C-2), 55.0 (C-1′), 41.1 (C-11), 34.5 (C-12). ESI-Orbitrap-MS 314.13837 [M + H]^+^ (−0.99 ∆ppm), C_18_H_19_NO_4_.

**Feruloyltryptamine (8)**. Light yellow powder. M.p. 148 °C. UV (MeOH, λ_max_, nm): 220, 290, 320. IR (water, ν_max_, cm^–1^): 3396, 2838, 1649, 1512, 1265. ^1^H NMR (600 MHz, CD_3_OD, δ in ppm, *J* in Hz): δ 3.03 (2H, t, *J* = 8.42 Hz, H-12), 3.62 (2H, t, *J* = 8.26 Hz, H-11), 3.90 (3H, s, OCH_3_), 6.43 (1H, d, *J* = 15.67 Hz, H-8), 6.81 (1H, d, *J* = 8.08 Hz, H-5), 7.02 (1H, td, *J* = 7.85 Hz, 7.27 Hz, 0.86 Hz, H-20), 7.04 (1H, dd, *J* = 8.23 Hz, 1.82 Hz, H-6), 7.09 (1H, dd, *J* = 8.24 Hz, 1.15 Hz, H-19), 7.11 (1H, dd, *J* = 14.88 Hz, 1.85 Hz, H-14), 7.14 (1H, d, *J* = 1.74 Hz, H-2), 7.35 (1H, d, *J* = 8.15 Hz, H-21), 7.45 (1H, d, *J* = 15.68 Hz, H-7), 7.60 (1H, d, *J* = 7.90 Hz, H-18). ^13^C NMR (150 MHz, CD_3_OD, δ in ppm): δ 167.8 (C-9), 148.5 (C-4), 147.9 (C-3), 140.5 (C-7), 136.9 (C-16), 127.4 (C-17), 122.0 (C-14), 121.8 (C-6), 120.9 (C-19), 118.2 (C-20), 117.5 (C-18), 115.1 (C-5), 111.9 (C-13), 110.8 (C-21), 110.1 (C-2), 55.0 (C-1′), 40.3 (C-11), 25.1 (C-12). ESI-Orbitrap-MS 337.15439 [M + H]^+^ (−0.83 ∆ppm), C_20_H_20_N_2_O_3_.

## 4. Conclusions

A protocol was developed to synthesize phenol amides in 25 h at room temperature, starting with a one hour pre-incubation of equimolar amounts of hydroxycinnamic acid and *N*,*N*′-dicyclohexylcarbodiimide in acetone, followed by a 24 h incubation with an equimolar amount of amine and aqueous sodium bicarbonate. Our protocol utilizes affordable and commercially available starting compounds and it is less laborious, easier and safer to use, and more sustainable than existing protocols for synthesis of poorly water-soluble amides.

To conclude, we present a general synthetic route that allows for the facile one-pot synthesis of structurally diverse phenol amides composed of combinations of various different hydroxycinnamic acids and amines. This protocol will facilitate future studies on the reactivity and structure-bioactivity relationships of this class of natural compounds.

## Figures and Tables

**Table 1:**
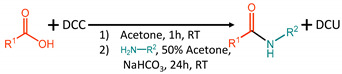
Amidation products, conversions, and yields.

Compound	Product	Name	Purity (%) ^a^	Conversion (%) ^b^	Yield (mg,%) ^c^
**1**	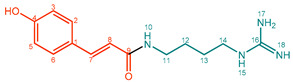	Coumaroylagmatine (CouAgm)	99%	26%	15.5 mg, 14%
**2**	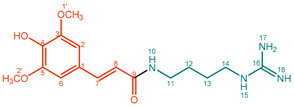	Sinapoylagmatine (SinAgm)	99%	9%	18.8 mg, 15%
**3**	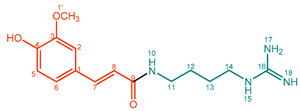	Feruloylagmatine (FerAgm)	95%	31%	19.4 mg, 19%
**4**	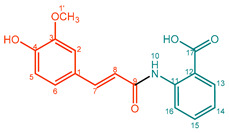	Feruloyl anthranilate (FerAnt*1*) ^d^	91%	22%	26.3 mg, 24%
**5**	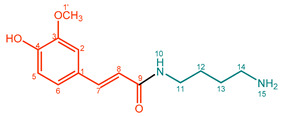	Feruloylputrescine (FerPut)	92%	33%	21.9 mg, 20%
**6**	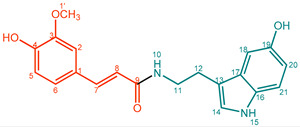	Feruloylserotonin (FerSrt)	77%	44%	26.7 mg, 21%
**7**	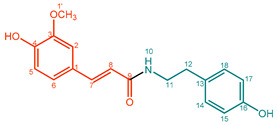	Feruloyltyramine (FerTrm)	86%	53%	24.5 mg, 21%
**8**	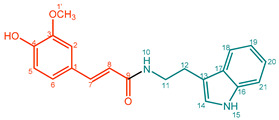	Feruloyltryptamine (FerTry)	88%	20%	23.1 mg, 20%

^a^ Determined using ^1^H NMR. ^b^ Determined based on the UV_320nm_ peak area of the phenol amide in samples taken before purification. Purified phenol amides from the synthesis were used as standards to quantify. ^c^ Based on the weight after freeze drying the purified compound and corrected for the purity of the obtained material. ^d^ Commonly called avenanthramide 1f.

## Data Availability

The data presented in this study are available on request from the corresponding author.

## Supplementary Materials

The following supporting information can be downloaded at: https://www.mdpi.com/article/10.3390/molecules27072203/s1, Detailed description of materials and analytical methods. Table S1: Conditions used and conversions obtained for the different optimisation protocols and conditions. Table S2: UHPLC-PDA-ESI-IT-MS data and ESI-Orbitrap-MS data for the isolated phenol amides. Figures S1–S8: RP-UHPLC-PDA-ESI-IT-MS UV-Vis and MS chromatograms of crude reaction mixtures of the individual phenol amides. Figure S9: UV–Vis spectra obtained for the isolated phenol amides. Figure S10: ATR-FTIR spectra obtained for the isolated phenol amides. Figure S11: DSC data obtained for the isolated phenol amides. Figures S13–S19: ^1^H and ^13^C NMR spectra for the isolated phenol amides.

## Sample Availability

Samples of the compounds are not available from the authors.

## References

[1] Roumani M., Besseau S., Gagneul D., Robin C., Larbat R. (2021). Phenolamides in plants: An update on their function, regulation, and origin of their biosynthetic enzymes. J. Exp. Bot..

[2] Bassard J.-E., Ullmann P., Bernier F., Werck-Reichhart D. (2010). Phenolamides: Bridging polyamines to the phenolic metabolism. Phytochemistry.

[3] Wang W., Snooks H.D., Sang S. (2020). The Chemistry and Health Benefits of Dietary Phenolamides. J. Agric. Food Chem..

[4] van Zadelhoff A., de Bruijn W.J., Fang Z., Gaquerel E., Ishihara A., Werck-Reichhart D.L., Zhang P., Zhou G., Franssen M.C., Vincken J.-P. (2021). Toward a systematic nomenclature for (neo) lignanamides. J. Nat. Prod..

[5] Roumani M., Duval R.E., Ropars A., Risler A., Robin C., Larbat R. (2020). Phenolamides: Plant specialized metabolites with a wide range of promising pharmacological and health-promoting interests. Biomed. Pharmacother..

[6] Procopio D., Siciliano C., Trombino S., Dumitrescu D.E., Suciu F., Di Gioia M.L. (2022). Green solvents for the formation of amide linkages. Org. Biomol..

[7] Collins F.W. (1989). Oat phenolics: Avenanthramides, novel substituted N-cinnamoylanthranilate alkaloids from oat groats and hulls. J. Agric. Food Chem..

[8] Miyagawa H., Ishihara A., Kuwahara Y., Ueno T., Mayama S. (1996). A stress compound in oats induced by victorin, a host-specific toxin from Helminthosporium victoriae. Phytochemistry.

[9] Negrel J., Smith T.A. (1984). Oxidation of *p*-coumaroylagmatine in barley seedling extracts in the presence of hydrogen peroxide or thiols. Phytochemistry.

[10] Yamazaki Y., Kawano Y., Uebayasi M. (2008). Induction of adiponectin by natural and synthetic phenolamides in mouse and human preadipocytes and its enhancement by docosahexaenoic acid. Life Sci..

[11] Todorovic M., Perrin D.M. (2020). Recent developments in catalytic amide bond formation. Peptide Sci..

[12] Jaradat D.S.M. (2018). Thirteen decades of peptide synthesis: Key developments in solid phase peptide synthesis and amide bond formation utilized in peptide ligation. Amino Acids.

[13] Santos A.S., Silva A.M., Marques M.M.B. (2020). Sustainable amidation reactions—Recent advances. Eur. J. Org. Chem..

[14] Fattahi N., Ayubi M., Ramazani A. (2018). Amidation and esterification of carboxylic acids with amines and phenols by N,N′-diisopropylcarbodiimide: A new approach for amide and ester bond formation in water. Tetrahedron.

[15] Stöekigt J., Zenk M. (1975). Chemical syntheses and properties of hydroxycinnamoyl-coenzyme A derivatives. Z. Naturforsch. C.

[16] Muroi A., Ishihara A., Tanaka C., Ishizuka A., Takabayashi J., Miyoshi H., Nishioka T. (2009). Accumulation of hydroxycinnamic acid amides induced by pathogen infection and identification of agmatine coumaroyltransferase in Arabidopsis thaliana. Planta.

[17] Alamgir K.M., Hojo Y., Christeller J.T., Fukumoto K., Isshiki R., Shinya T., Baldwin I.T., Galis I. (2016). Systematic analysis of rice (Oryza sativa) metabolic responses to herbivory. Plant Cell Environ..

[18] Pathak G., Das D., Rokhum L. (2016). A microwave-assisted highly practical chemoselective esterification and amidation of carboxylic acids. RSC Adv..

[19] Lenstra D.C., Rutjes F.P., Mecinović J. (2014). Triphenylphosphine-catalysed amide bond formation between carboxylic acids and amines. Chem. Commun..

[20] Dalu F., Scorciapino M.A., Cara C., Luridiana A., Musinu A., Casu M., Secci F., Cannas C. (2018). A catalyst-free, waste-less ethanol-based solvothermal synthesis of amides. Green Chem..

[21] Lenstra D.C., Nguyen D.T., Mecinović J. (2015). Zirconium-catalyzed direct amide bond formation between carboxylic esters and amines. Tetrahedron.

[22] Pihlava J.-M. (2014). Identification of hordatines and other phenolamides in barley (*Hordeum vulgare*) and beer by UPLC-QTOF-MS. J. Cereal Sci..

[23] Dimberg L.H., Sunnerheim K., Sundberg B., Walsh K. (2001). Stability of oat avenanthramides. Cereal Chem..

[24] Hwang J.T., Kim Y., Jang H.-J., Oh H.-M., Lim C.-H., Lee S.W., Rho M.-C. (2016). Study of the UV light conversion of feruloyl amides from Portulaca oleracea and their inhibitory effect on IL-6-induced STAT3 activation. Molecules.

