# Supplementary material for: Facile Amidation of Non-Protected Hydroxycinnamic Acids for the Synthesis of Natural Phenol Amides
Source: Molecules. 2022 Mar 28;27(7):2203. doi: 10.3390/molecules27072203 (PMC9000787; doi:10.3390/molecules27072203)
Supplement: Supplementary file 1 [file molecules-27-02203-s001.zip › molecules-1649245-supplementary.pdf]

Facile amidation of non-protected hydroxycinnamic acids for the synthesis of natural phenol amides

Annemiek van Zadelhoff, Jean-Paul Vincken, and Wouter J. C. de Bruijn

Detailed description of materials and analytical methods

Materials

Ferulic acid (99 %), serotonin hydrochloride (98 %), anthranilic acid (99.5 %), tyramine (99 %), tryptamine (97 %), putrescine (98.5 %), and sodium bicarbonate (99 %) were purchased from Sigma-Aldrich (Saint Louis, Missouri, USA). *N*,*N*'-dicyclohexylcarbodiimide (99 %) and agmatine sulphate (97 %) were purchased from Fisher Scientific B.V. (Hampton, New Hampshire, USA). *N*,*N*'-diisopropylcarbodiimide (DIC) was purchased from Merck Life Science N.V. (Darmstadt, Germany). Acetone, ethyl acetate, ULC-MS grade ACN and water, both acidified with 0.1 % formic acid (FA), formic acid, and glacial acetic acid were purchased from Biosolve B.V. (Valkenswaard, The Netherlands). Water for other purposes than UHPLC-MS was purified using a Milli-Q purification system equipped with a 0.22 µm filter (Millipore, Burlington, Massachusetts, USA).

Purification by flash chromatography

Purified phenol amides were obtained by using UV-directed fraction collection after separation by reversed phase flash chromatography on a Pure C-850 FlashPrep system (Büchi, Flawil, Switzerland) operated in flash mode. Separation was performed on a 12 g FlashPure ID C18 column (Büchi). The eluents used were water with 1 % (*v*/*v*) FA (eluent A) and ACN with 1 % (*v*/*v*) FA (eluent B). Fractions were collected based on absorbance at 290, 300, 310, and 320 nm. The elution programme used for feruloyltyramine, feruloyltryptamine, and feruloyl anthranilate was 2 column volumes (CV) isocratic at 15 % B, 30 CVs linear gradient to 45 % B, 1 CV linear gradient to 100 % B, and 5 CVs isocratic at 100 % B. For purification of coumaroylagmatine, sinapoylagmatine, feruloylagmatine, feruloylputrescine, and feruloylserotonin the elution programme used was 2 CVs isocratic at 5 % B, 20 CVs linear gradient to 25 % B, 1 CV linear gradient to 100 % B, and 5 CVs isocratic at 100 % B. After collecting and pooling the fractions containing the desired product, ACN was evaporated under reduced pressure at 40 °C. The remaining water was removed by freeze drying.

UHPLC-PDA-ESI-IT-MS analysis

For analysis of the phenol amides, the samples were separated on a Thermo Vanquish UHPLC system (Thermo Scientific, San Jose, CA) equipped with a pump, degasser, autosampler, and PDA detector. The flow rate was set at 400 µL/min at a column temperature of 35 °C. The PDA detector was set to measure wavelengths in a range of 190-680 nm. Water (A) and ACN (B), both acidified with 0.1% (*v*/*v*) FA, were used as eluents. Samples (1 µL) were injected on a Waters Acquity BEH C18 column (150 mm × 2.1 mm i.d., 1.7 µm particle size) with a VanGuard guard column of the same material (5 mm × 2.1 mm i.d., 1.7 µm particle size) (Waters, Milford, MA, USA). The following elution programme was used: isocratic at 10 % B for 1.09 min, 1.09-19.26 min linear gradient to 60 % B, 19.26-20.35 min linear gradient to 100 % B and isocratic at 100 % B from 20.35-25.80 min. The eluent was adjusted to its starting composition in 1.09 min, followed by equilibration for 5.45 min.

Mass spectrometric data were acquired using a LTQ Velos Pro linear ion trap mass spectrometer (Thermo Scientific) equipped with a heated ESI probe coupled in-line to the RP-UHPLC system. Nitrogen was used both as sheath gas (50 arbitrary units) and auxiliary gas (13 arbitrary units). Data was collected in positive and negative ionisation mode over the *m*/*z* range 190-1500. MS^2 analysis was performed by collision-induced dissociation (CID) with a normalised collision energy of 35 %. The MS^2 fragmentation was performed on the most abundant ions in full MS. Dynamic exclusion, with a repeat count of 4, a repeat duration of 5.0 s and an exclusion duration of 5.0 s, was used to obtain MS^2 spectra of multiple different ions present in full MS at the same time. Most settings were optimised via

automatic tuning using LTQ Tune Plus (Xcalibur version 4.1, Thermo Scientific). The capillary temperature was 263 °C; the probe heater temperature was 425 °C; the source voltage was 2.5 kV in negative mode and 3.5 kV in positive mode; and the S-lens RF level was 67.63 % in positive mode and 62.64 % in negative mode. Data processing was performed using Xcalibur 4.1 (Thermo Scientific).

NMR spectroscopy

Prior to analysis, 1.5 mg of phenol amide was dissolved in 500 µL deuterated methanol (Sigma-Aldrich). NMR spectra were recorded at a probe temperature of 300 K on a Bruker Avance-III-600 spectrometer (Bruker, Billerica, MA, USA) located at the MAGNEtic resonance research FacilitY of Wageningen University. For all compounds, 1D ^{1}H and ^{13}C and 2D HMBC and HMQC spectra were acquired. Data processing was performed using TopSpin 4.1.1 (Bruker).

High resolution ESI-Orbitrap-MS

High-resolution mass spectra were recorded on a Q Exactive Focus hybrid quadrupole-Orbitrap mass spectrometer (Thermo Scientific), equipped with a heated ESI probe, coupled to a Vanquish UHPLC system. The UHPLC system, system parameters, eluents, and elution program used were identical to those described for UHPLC-PDA-ESI-IT-MS. Full MS data were recorded in both negative and positive ionisation mode over a range of *m/z* 200-1,000 at a resolution of 70,000. The mass spectrometer was calibrated in both positive and negative ionisation mode using Tune 2.11 software (Thermo Scientific) by direct infusion of Pierce LTQ ESI positive and negative ion calibration solutions (Thermo Scientific). Nitrogen was used as sheath gas (46.7 arbitrary units) and auxiliary gas (10.8 arbitrary units). The capillary temperature was 254 °C; the probe heater temperature was 408 °C; the source voltage was 2.5 kV in negative mode and 3.5 kV in positive mode; and the S-lens RF level was 50 %. MS^2 analysis was performed by higher-energy collisional dissociation (HCD) with a normalised collision energy of 30%. The MS^2 fragmentation was performed on the most abundant ions in full MS and on ions specified in an inclusion list, containing the phenol amide masses. Dynamic exclusion settings were automated by Tune 2.11 software, to obtain MS^2 spectra of multiple different ions present in full MS at the same time. Data processing was performed using Xcalibur 4.1 (Thermo Scientific).

UV-Vis spectroscopy

UV-Vis spectra were recorded using a Genesis 150 UV/Vis spectrophotometer (Thermo Scientific). Solutions of 0.01 mg/mL in 1 mL quartz cuvettes were measured over a range of 190 to 1100 nm.

ATR-FTIR spectrometry

Attenuated total reflectance Fourier transform infrared (ATR-FTIR) spectra were recorded using an Invenio-S (Bruker, Billeria, MA, USA) equipped with a BioATRcell II (Harricks, Pleasantville, NY, USA). Independent duplicates were averaged using OPUS software (Bruker). Samples with a concentration of 5 mg/mL in water were analysed over a wavelength range of 700 to 4000 nm at a controlled temperature of 25 °C using 64 scans and a resolution of 4 cm^{-1}.

DSC

Melting points were determined using a Q200 differential scanning calorimeter (DSC) (TA Instruments, New Castle, DE, USA) over a temperature range of 10 to 250 °C with a slope of 2 °C/min, instant cooling, and a second heating slope using the same conditions as the first. Data of independent duplicates was analysed using Universal V4.5A software (TA Instruments).

Optimisation protocols and conditions

Table S1 Conditions used and conversions obtained for the different optimisation protocols and conditions. All optimisation experiments were performed on a 2 mL Eppendorf scale and were continuously stirred during incubation by using an Eppendorf shaker. The yield was determined based on the UV320nm peak area derived from the RP-UHPLC-PDA-ESI-IT-MS data.

	**Protocol 1: Two-step protocol** [a]		**Protocol 2: One-step reaction** [b]						**Protocol 3: 1 h pre-incubation of hydroxycinnamic acid and DCC** [c]	
	Condition 1	*Condition 2*	*Condition 3*	*Condition 4*	*Condition 5*	*Condition 6*	*Condition 7*	*Condition 8*	*Condition 9*	*Condition 10*
	24 hours incubation	48 hours incubation	24 hours incubation	48 hours incubation	No *N*-hydroxy-succinimide	4 molar eq. of $NaHCO_3$	No $NaHCO_3$, 2 molar eq. of NaOH	2 molar eq. of amine	Absence of $NaHCO_3$	Equimolar amount of $NaHCO_3$
**Product**	Conversion based on UV_{320nm} (%)									
**FerAgm**	12.3	19.0	1.0	1.1	n.t.[d]	n.t.	n.t.	n.t.	4.6	29.7
**FerAnt*1***	1.7	2.2	0.6	0.6	n.t.	n.t.	n.t.	n.t.	4.2	5.7
**FerPut**	8.0	10.8	0.2	0.4	n.t.	n.t.	n.t.	n.t.	n.t.	n.t.
**FerSrt**	9.3	9.2	0.8	0.8	n.t.	n.t.	n.t.	n.t.	n.t.	n.t.
**FerTrm**	12.3	15.5	1.1	1.1	1.7	5.0	0.2	8.4	26.9	43.8
**FerTry**	3.4	3.2	1.1	1.5	0.1	0.6	0	0.2	11.4	10.0

[a] Based on the protocols of Stöeckigt and Zenk (1975) [15], Negrel *et al*. (1984) [9], and Muroi *et al*. (2009) [16]. Incubation of equimolar amounts of hydroxycinnamic acid, *N*-hydroxysuccinimide, and DCC in ethyl acetate for 24 hours, followed by filtration to remove the insoluble DCU formed. Ethyl acetate was removed under reduced pressure. The ester formed was dissolved in acetone and was incubated for 24 or 48 hours with an equimolar amount of amine and 2.5 molar equivalents of aqueous $NaHCO_3$.

[b] Equimolar amounts of hydroxycinnamic acid, amine, DCC, and *N*-hydroxysuccinimide were dissolved in acetone. $NaHCO_3$ dissolved in water was added in a 2.5 molar equivalent of the other compounds used. The mixture was incubated for 24 hours, unless stated otherwise.

[c] Equimolar amounts of hydroxycinnamic acid and DCC were dissolved in acetone and incubated for 1 hour. To this solution an equimolar amount of amine dissolved in water was added. The mixture was incubated for 24 hours.

[d] n.t. = not tested

UHPLC-PDA-ESI-IT-MS

Table S2 UHPLC-PDA-ESI-IT-MS data and ESI-Orbitrap-MS data for the synthesized phenol amides. Only fragments with a relative abundance above 10% are given.

	UHPLC-PDA-ESI-IT-MS				ESI-Orbitrap-MS				
**Compound**	**RT (min)**	**λ_{max} (nm)**	**$[M+H]^+$ (*m/z*)**	**MS² (*m/z*) (relative intensity)[a]**	**Molecular formula**	**$[M+H]^+$ (*m/z*) Calculated**	**Observed**	**Error (ppm)**	**MS² (*m/z*) (relative intensity)[b]**
Coumaroylagmatine (1)	5.43	198, 294	277	260 (100), 114 (20), 217 (18), 261 (16), 218 (10), 115 (10)	$C_{14}H_{20}N_4O_2$	277.16590	277.16559	-1.13	147.04382 (100), 119.04921 (12), 148.04715 (11)
Sinapoylagmatine (2)	6.27	202, 318	337	320 (100), 207 (58), 321 (17), 114 (17), 303 (13)	$C_{16}H_{24}N_4O_3$	337.18703	337.18701	-0.06	207.06493 (100), 175.03876 (69), 147.04385 (14), 208.06822 (13), 114.10275 (11)
Feruloylagmatine (3)	6.05	202, 321	307	290 (100), 176 (28), 114 (19), 291 (18), 248 (11)	$C_{15}H_{22}N_4O_3$	307.17647	307.17630	-0.55	177.05447 (100), 145.02835 (48), 178.05785 (11)
Feruloylputrescine (5)	5.00	205, 318	265	248 (100), 177 (95), 206 (15), 114 (11)	$C_{14}H_{20}N_2O_3$	265.15467	265.15442	-0.94	145.02832 (100), 177.05446 (43), 117.03365 (27), 72.08147 (13), 149.05965 (12), 146.03169 (11)
Feruloylserotonin (6)	10.71	202, 314	353	177 (100)	$C_{20}H_{20}N_2O_4$	353.14958	353.14938	-0.57	177.05449 (100), 145.02834 (95), 117.03372 (11), 160.07559 (10), 178.05779 (10)
Feruloyltyramine (7)	11.06	210, 318	314	177 (100)	$C_{18}H_{19}NO_4$	314.13868	314.13837	-0.99	145.02835 (100), 121.06497 (98), 177.05453 (62), 117.03373 (16), 93.07033 (15), 146.03166 (11)
Feruloyltryptamine (8)	14.60	290, 318	337	177 (100)	$C_{20}H_{20}N_2O_3$	337.15467	337.15439	-0.83	145.02841 (100), 177.05450 (93), 117.03365 (16), 144.08105 (16), 273.90131 (13), 178.05759 (12)
**Compound**	**RT (min)**	**λ_{max} (nm)**	**$[M-H]^-$ (*m/z*)**	**MS² (*m/z*) (relative intensity)**	**Molecular formula**	**$[M-H]^-$ (*m/z*) Calculated**	**Observed**	**Error (ppm)**	**MS² (*m/z*) (relative intensity)**
Feruloyl anthranilate (4)	15.09	210, 338	312	268 (100), 162 (25), 175 (18)	$C_{17}H_{15}NO_5$	312.08775	312.08768	-0.22	252.06630 (100), 134.03572 (33), 253.07042 (20), 144.04416 (17), 224.07111 (16), 160.01532 (12)

[a] Fragments obtained from collision-induced dissociation (CID) fragmentation. [b] Fragments obtained from higher energy collisional dissociation (HCD) fragmentation.

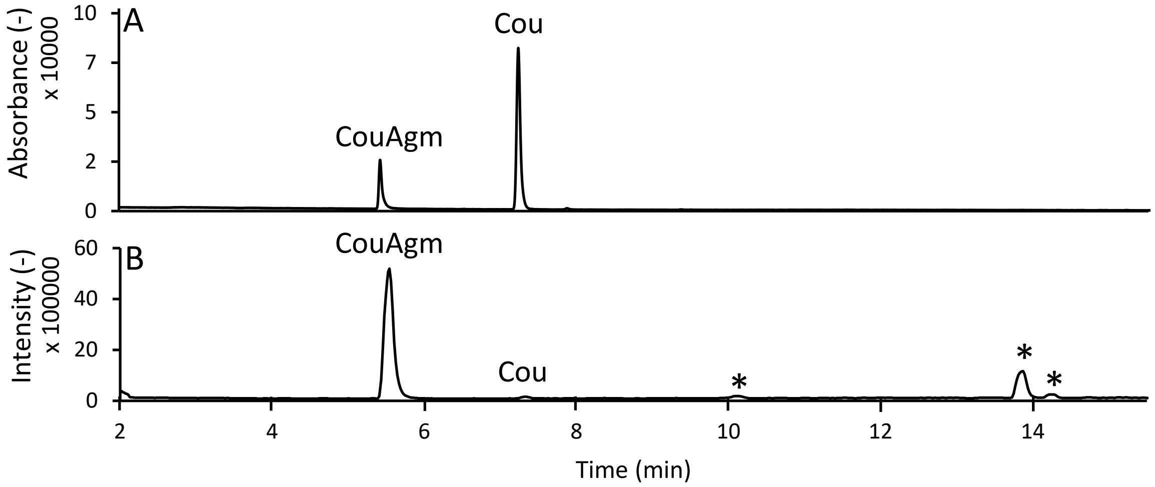

*Figure S1 RP-UHPLC-PDA-ESI-IT-MS data of coumaroylagmatine; UV-Vis chromatogram at 320 nm (A) and MS base peak chromatogram in positive ionisation (B). * represent system peaks.*

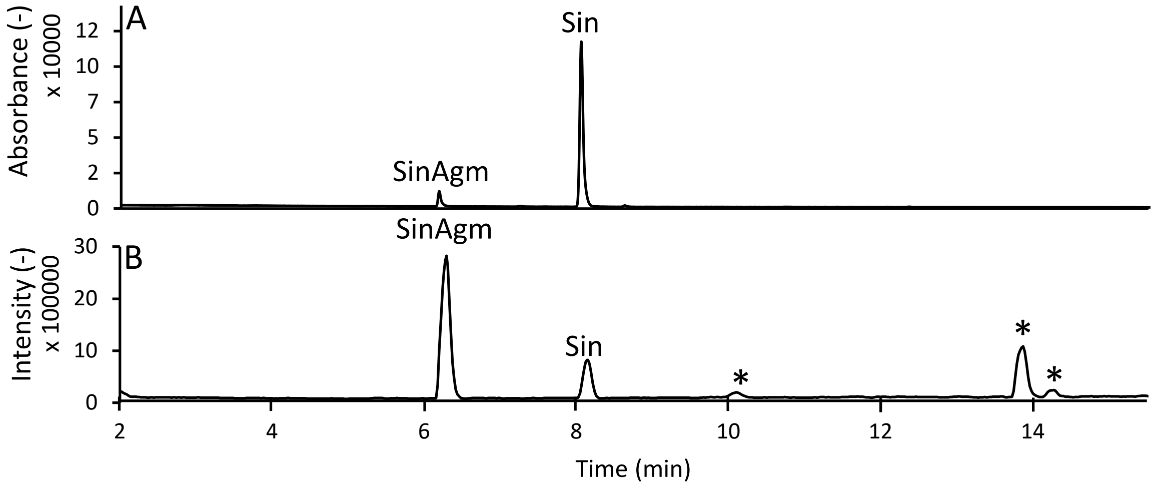

*Figure S2 RP-UHPLC-PDA-ESI-IT-MS data of sinapoylagmatine; UV-VIS chromatogram at 320 nm (A) and MS base peak chromatogram in positive ionisation (B). * represent system peaks.*

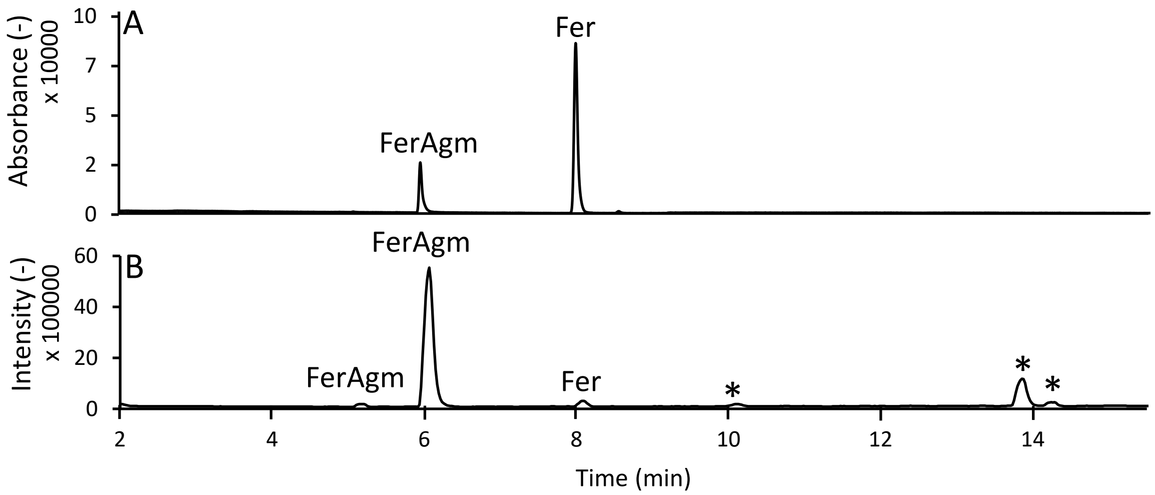

*Figure S3 RP-UHPLC-PDA-ESI-IT-MS data of feruloylagmatine; UV-VIS chromatogram at 320 nm (A) and MS base peak chromatogram in positive ionisation (B). * represent system peaks.*

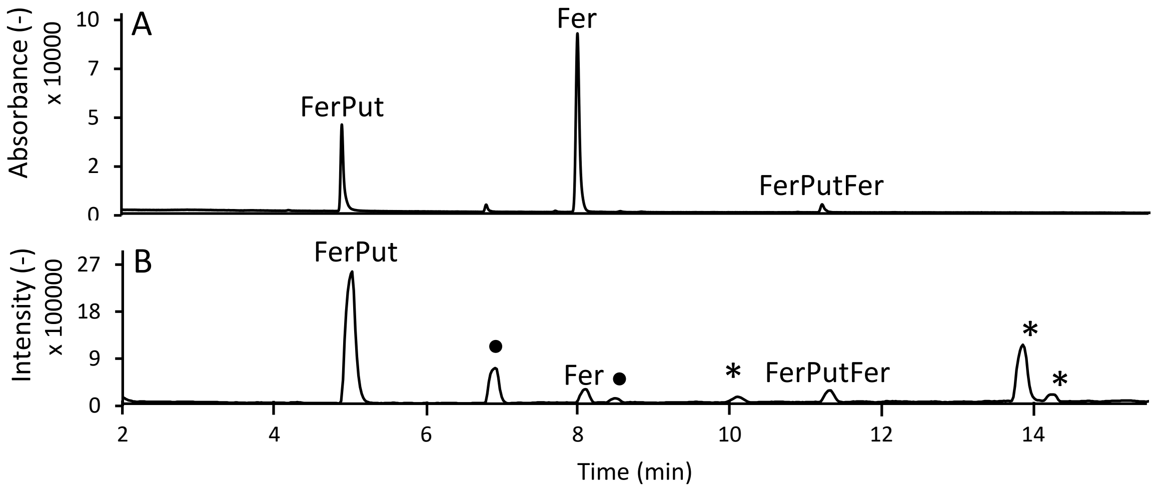

*Figure S4 RP-UHPLC-PDA-ESI-IT-MS data of feruloylputrescine; UV-VIS chromatogram at 320 nm (A) and MS base peak chromatogram in positive ionisation (B). * represent system peaks and • represent unidentified by-products.*

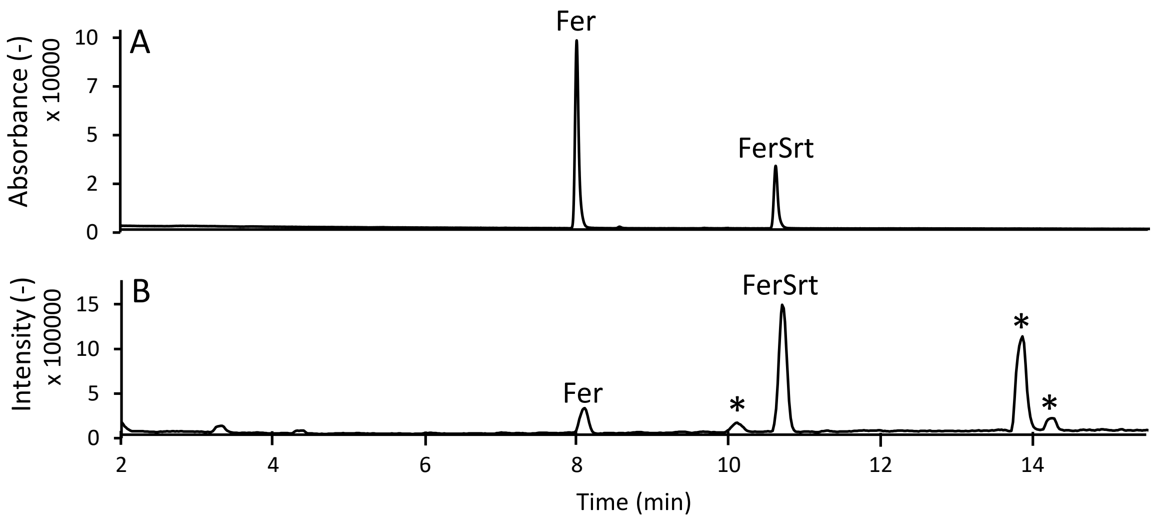

*Figure S5 RP-UHPLC-PDA-ESI-IT-MS data of feruloylserotonin; UV-VIS chromatogram at 320 nm (A) and MS base peak chromatogram in positive ionisation (B). * represent system peaks.*

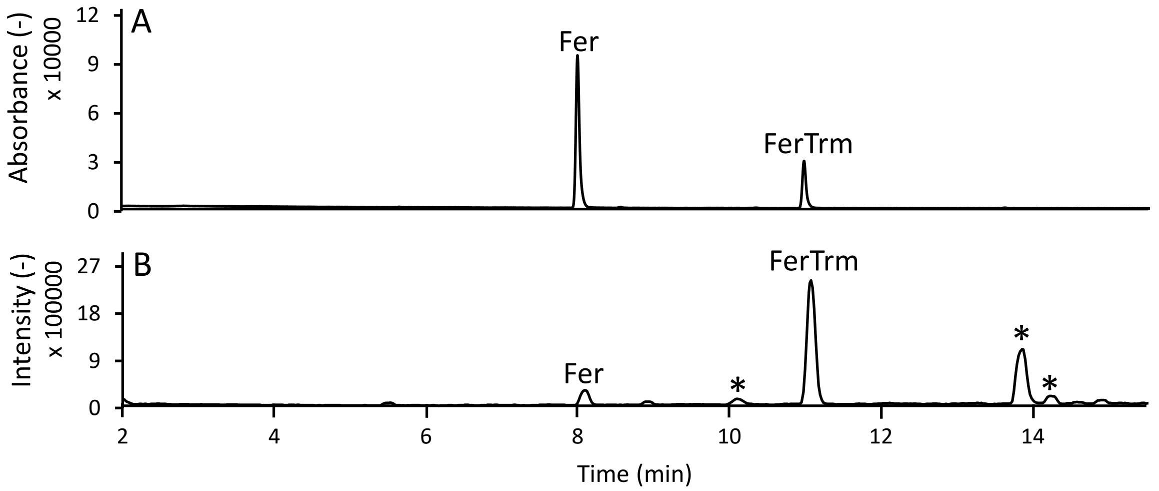

*Figure S6 RP-UHPLC-PDA-ESI-IT-MS data of feruloyltyramine; UV-VIS chromatogram at 320 nm (A) and MS base peak chromatogram in positive ionisation (B). * represent system peaks.*

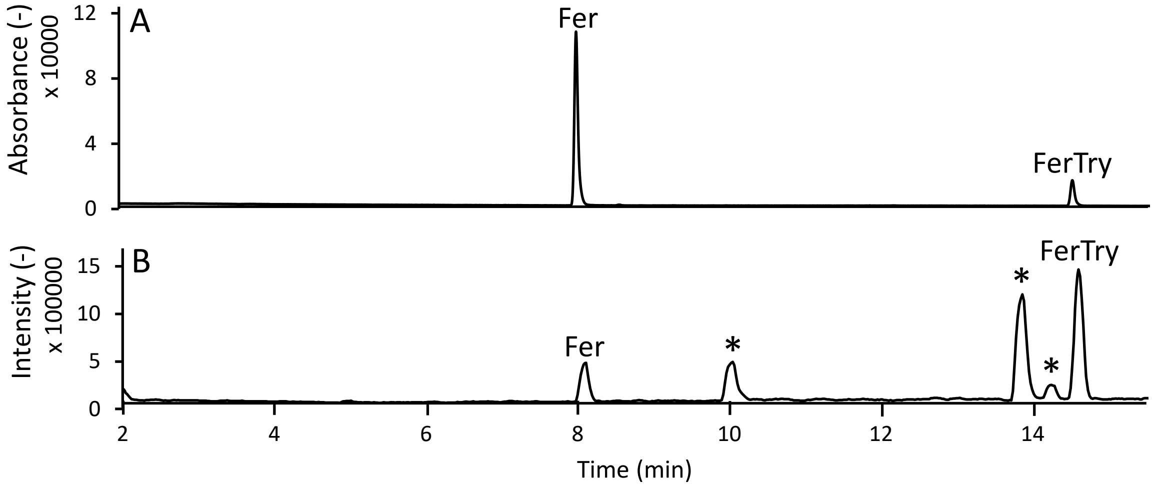

*Figure S7 RP-UHPLC-PDA-ESI-IT-MS data of feruloyltryptamine; UV-VIS chromatogram at 320 nm (A) and MS base peak chromatogram in positive ionisation (B). * represent system peaks.*

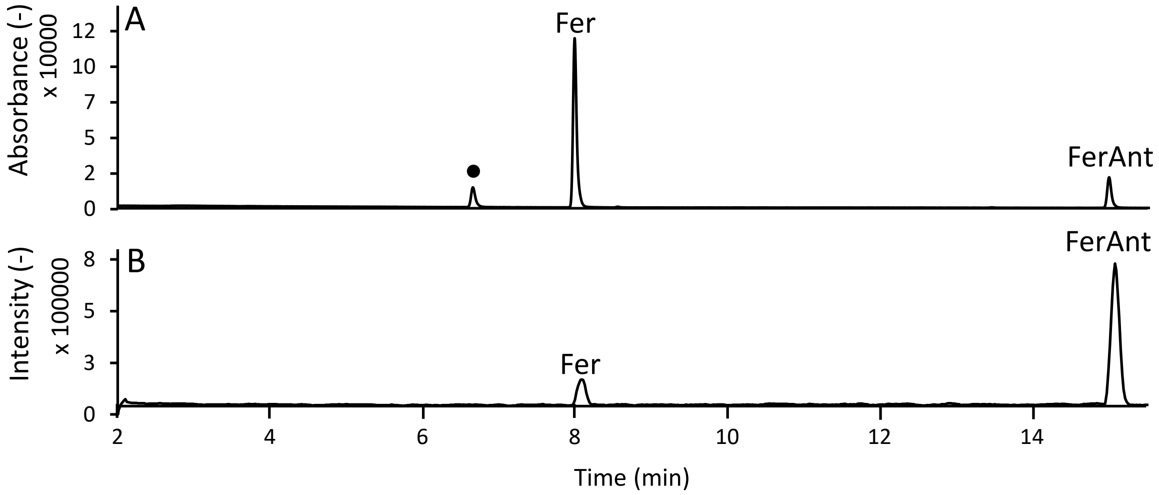

Figure S8 RP-UHPLC-PDA-ESI-IT-MS data of feruloyl anthranilate; UV-VIS chromatogram at 320 nm (A) and MS base peak chromatogram in negative ionisation (B). • represents unidentified by-products.

UV-Vis spectra

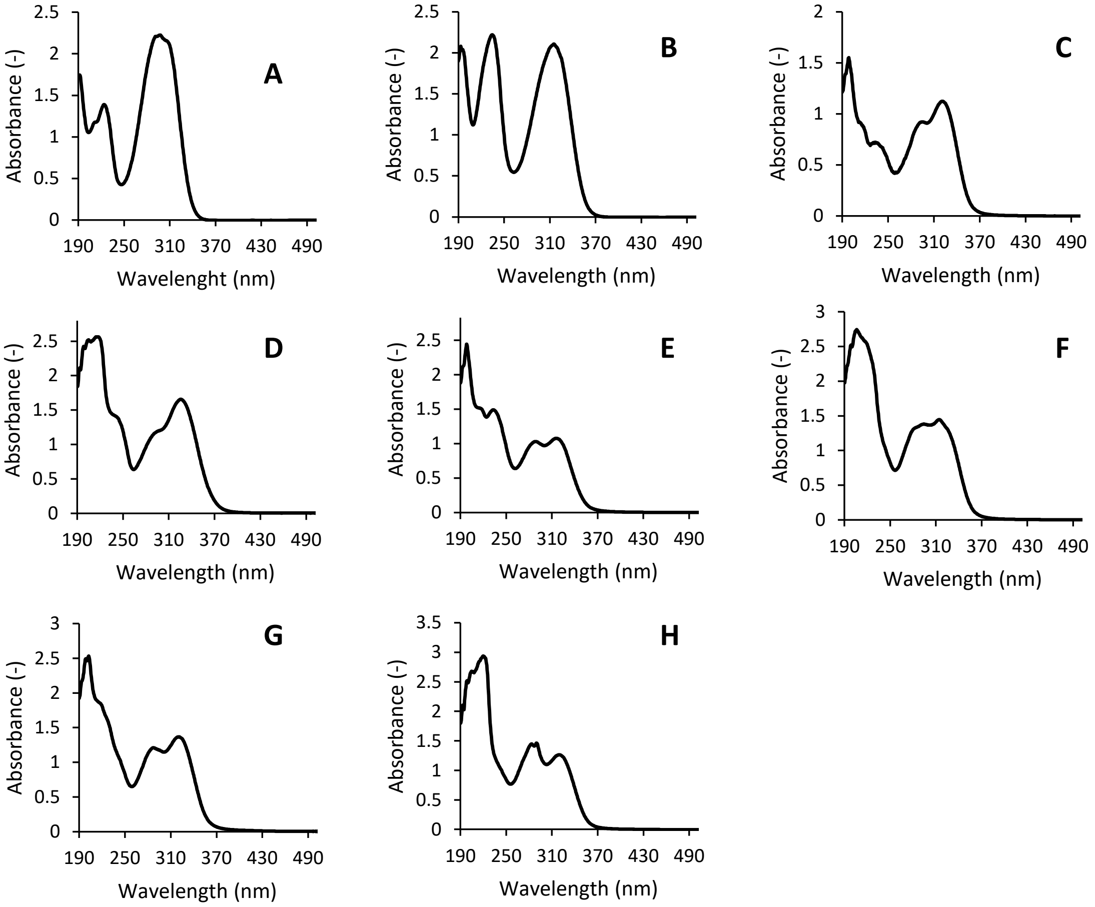

Figure S9 UV-Vis spectra obtained for CouAgm (A), SinAgm (B), FerAgm (C), FerAnt1 (D), FerPut (E), FerSrt (F), FerTrm (G), and FerTry (H). Data shown is the average of independent duplicates.

IR spectra

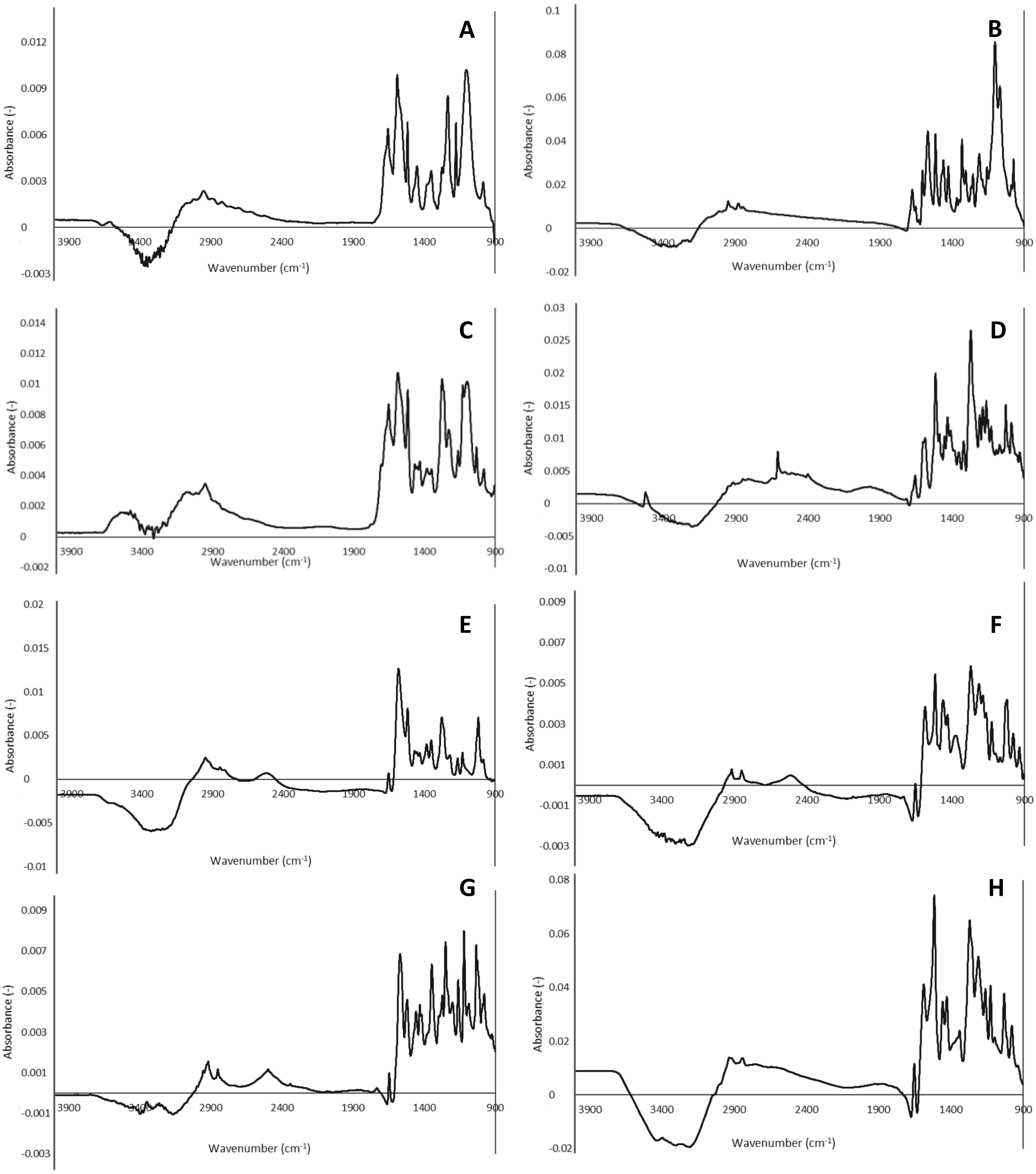

Figure S10 ATR-FTIR spectra obtained for CouAgm (A), SinAgm (B), FerAgm (C), FerAnt (D), FerPut (E), FerSrt (F), FerTrm (G), and FerTry (H). Data shown is the average of independent duplicates.

Melting points

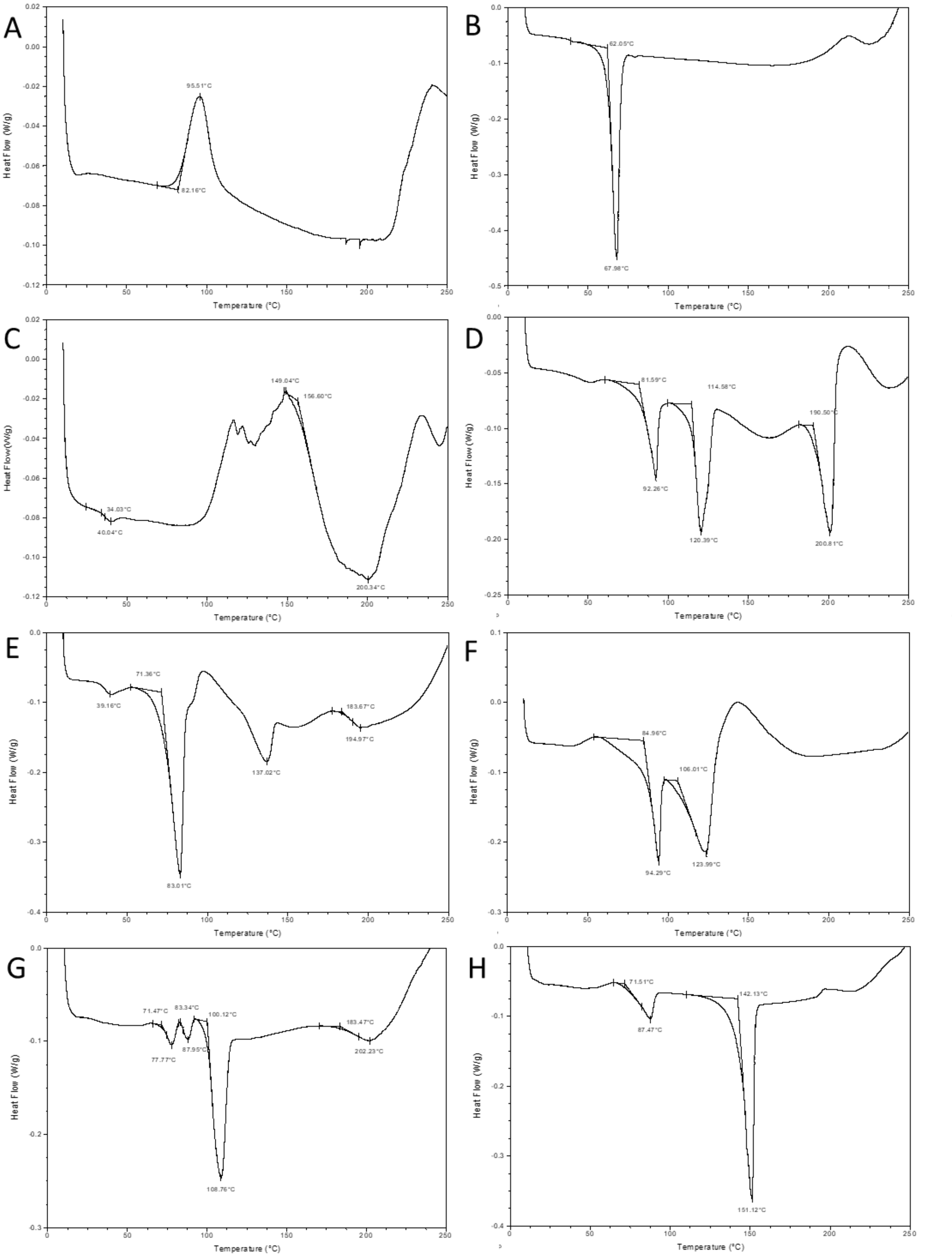

Figure S11 DSC data obtained for CouAgm (A), SinAgm (B), FerAgm (C), FerAnt (D), FerPut (E), FerSrt (F), FerTrm (G), and FerTry (H).

NMR spectra

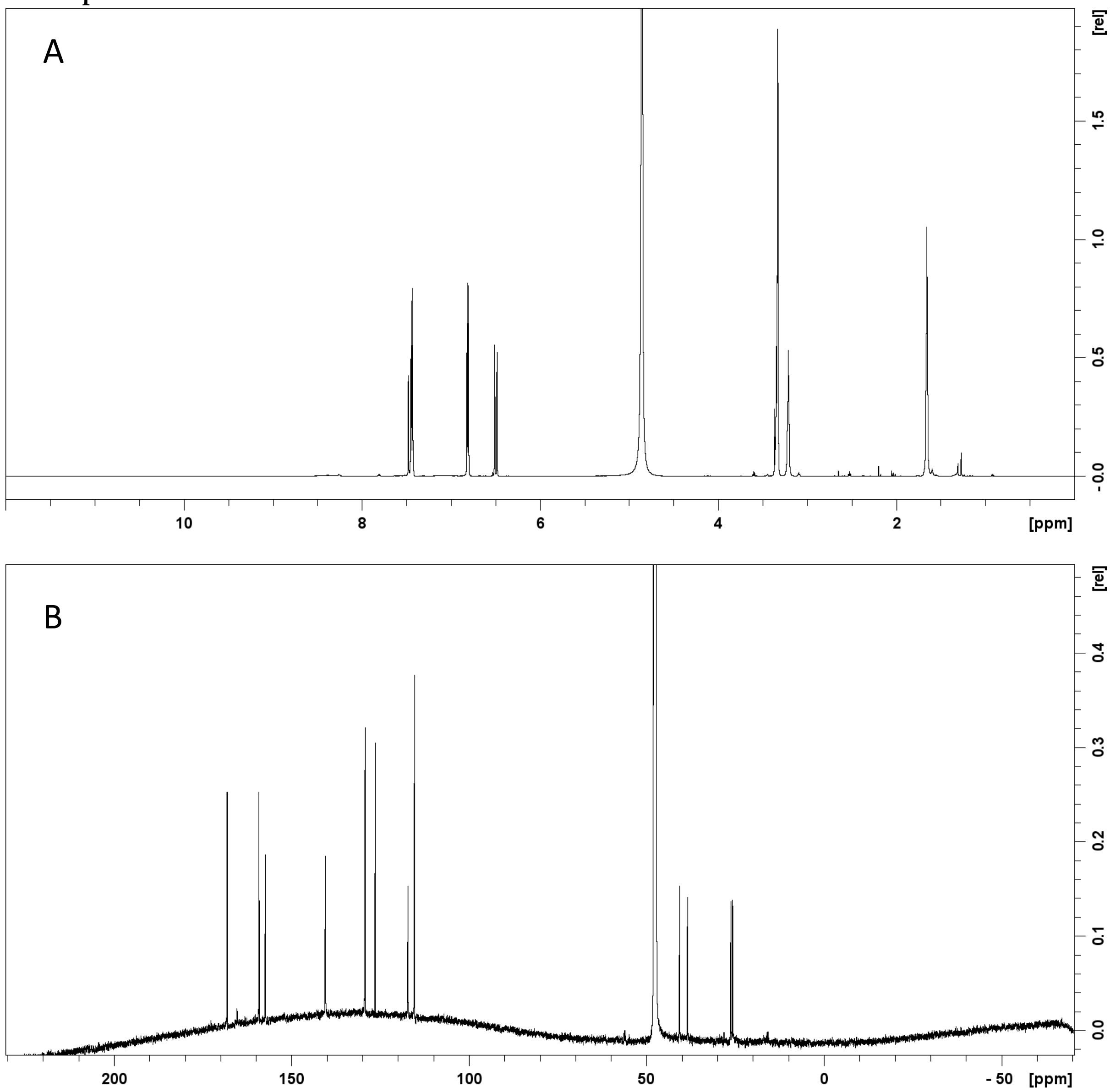

Figure S12 1H (A) and 13C (B) NMR spectra for CouAgm

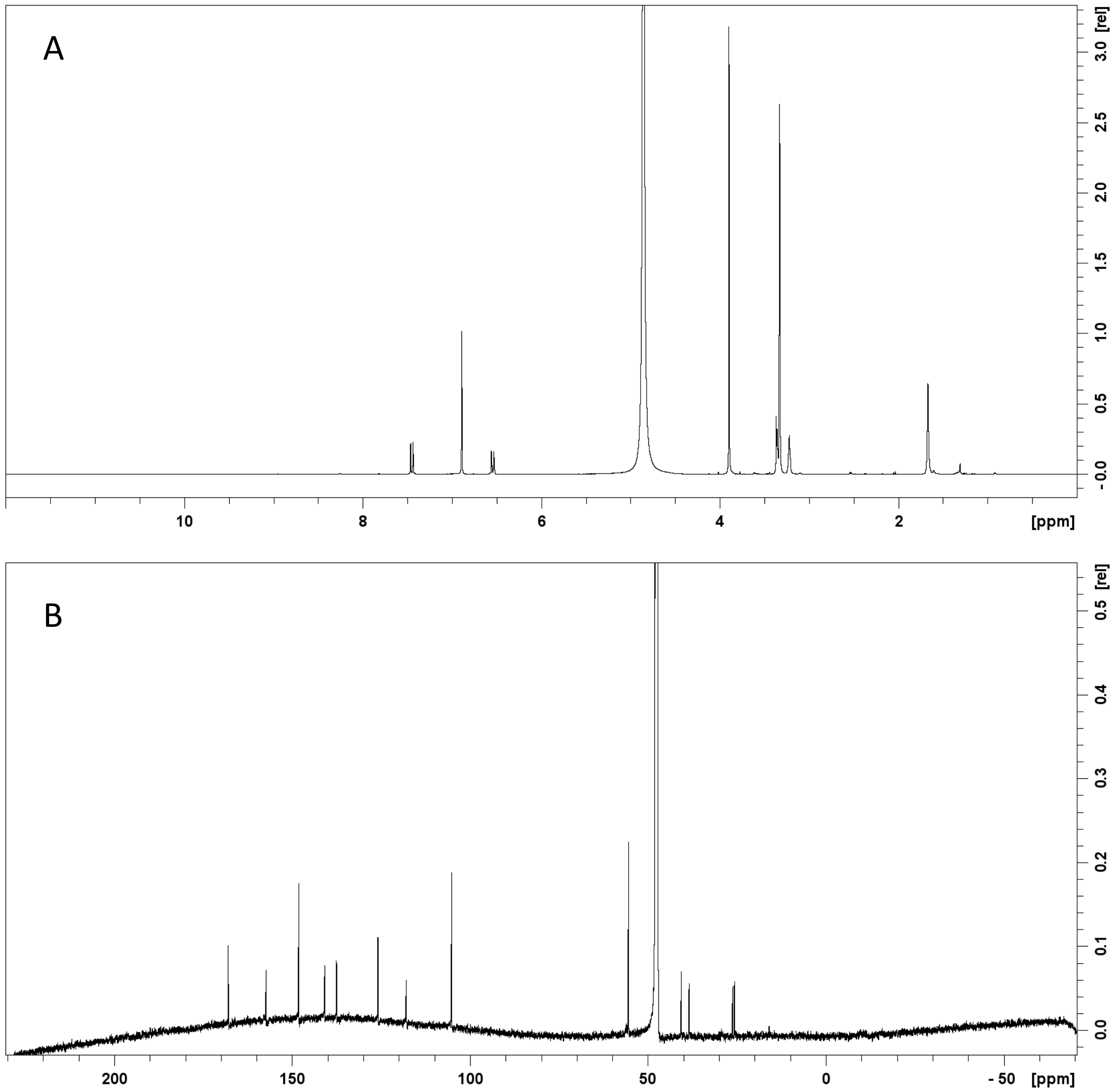

Figure S13 1H (A) and 13C (B) NMR spectra for SinAgm

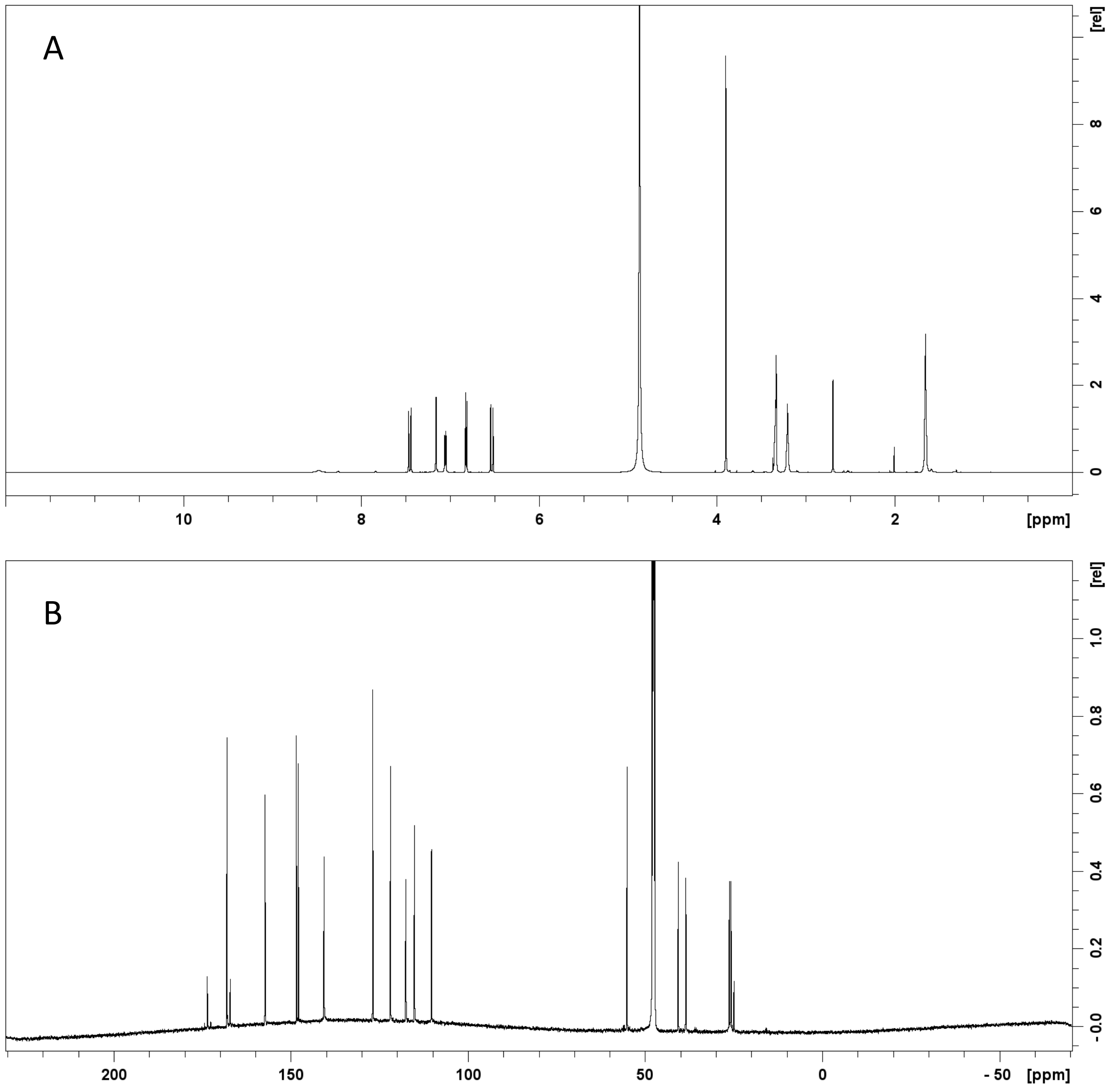

Figure S14 1H (A) and 13C (B) NMR spectra for FerAgm

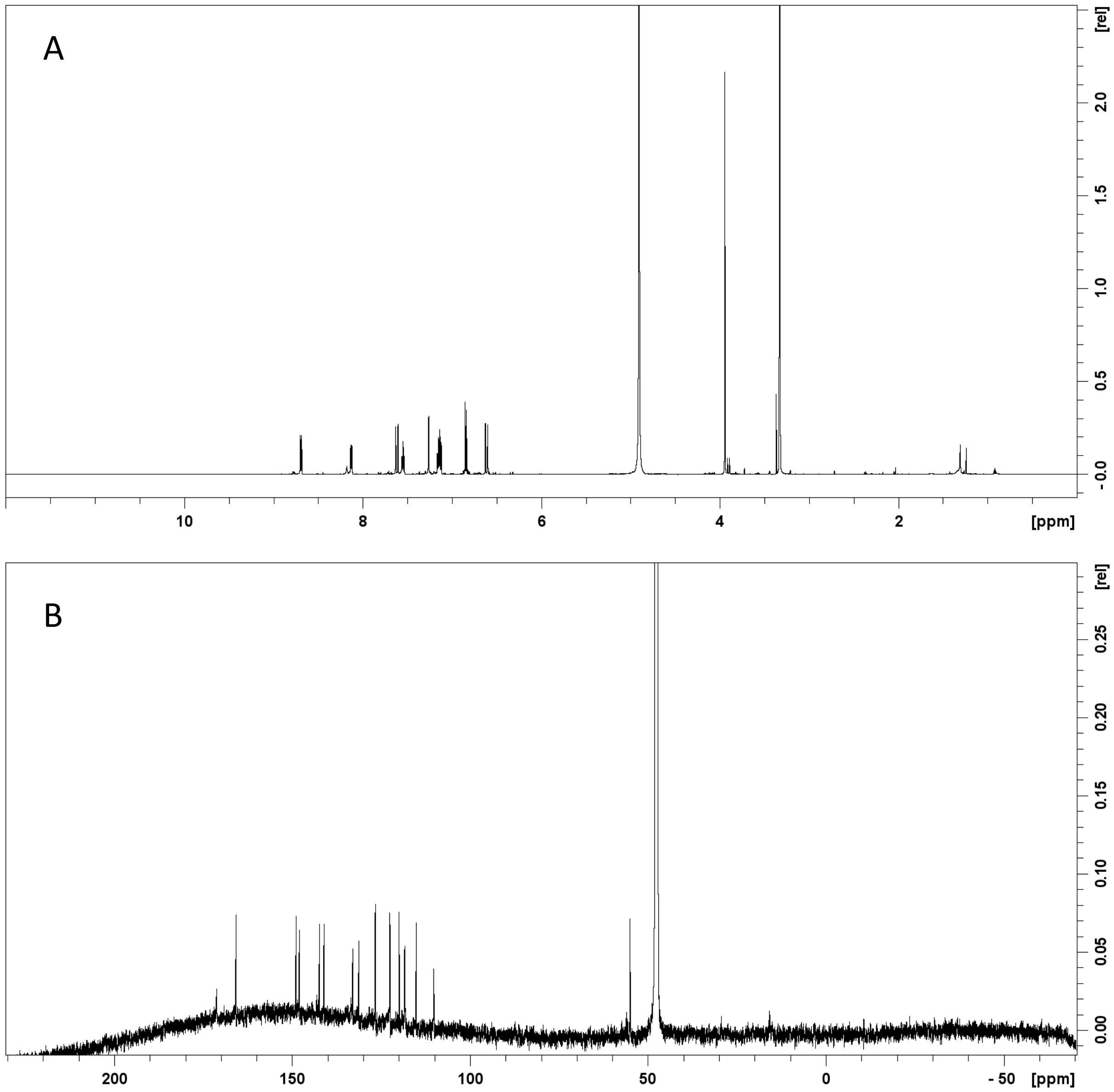

Figure S15 1H (A) and 13C (B) NMR spectra for FerAnt

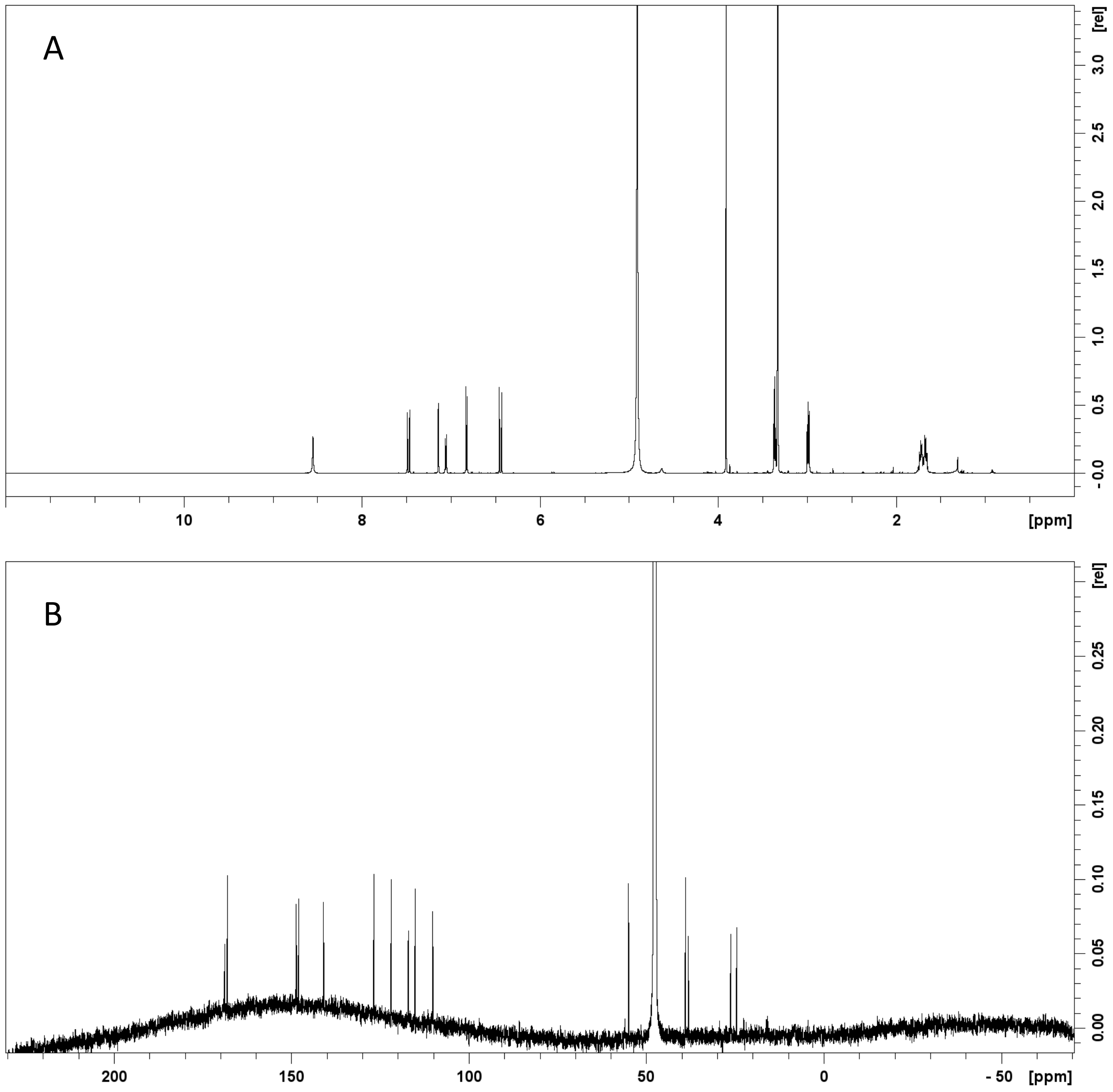

Figure S16 1H (A) and 13C (B) NMR spectra for FerPut

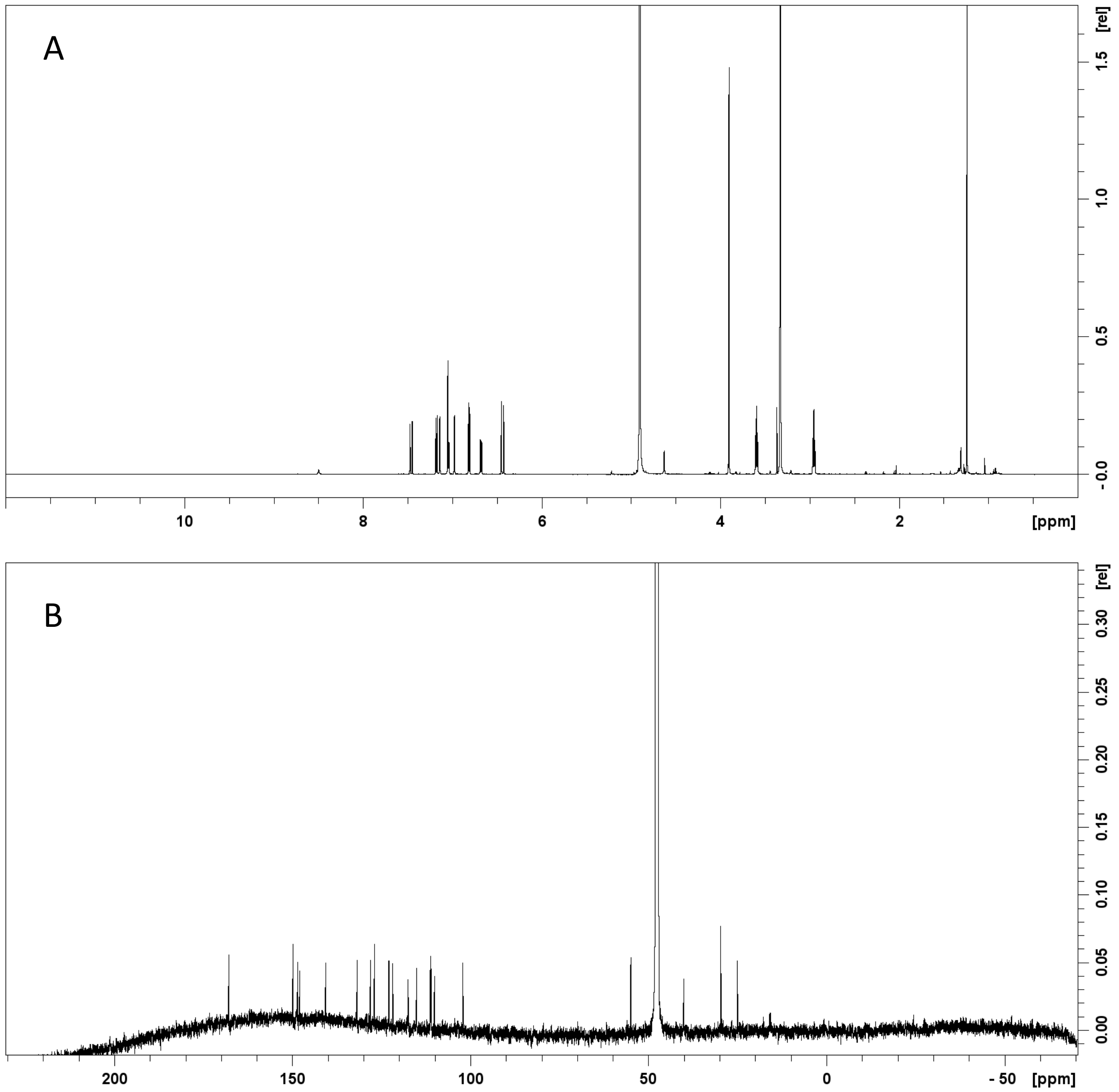

Figure S17 1H (A) and 13C (B) NMR spectra for FerSrt

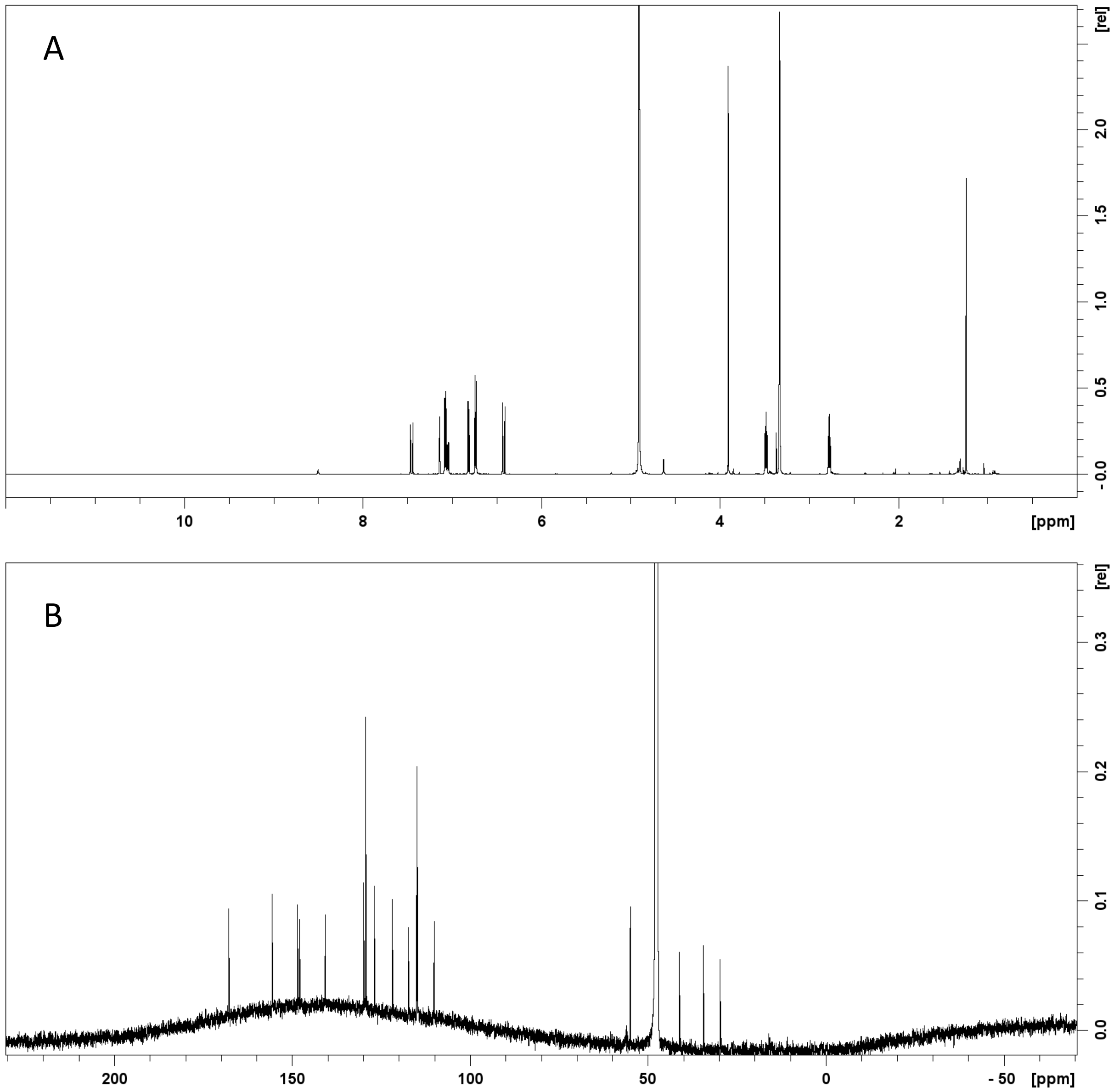

Figure S18 1H (A) and 13C (B) NMR spectra for FerTrm

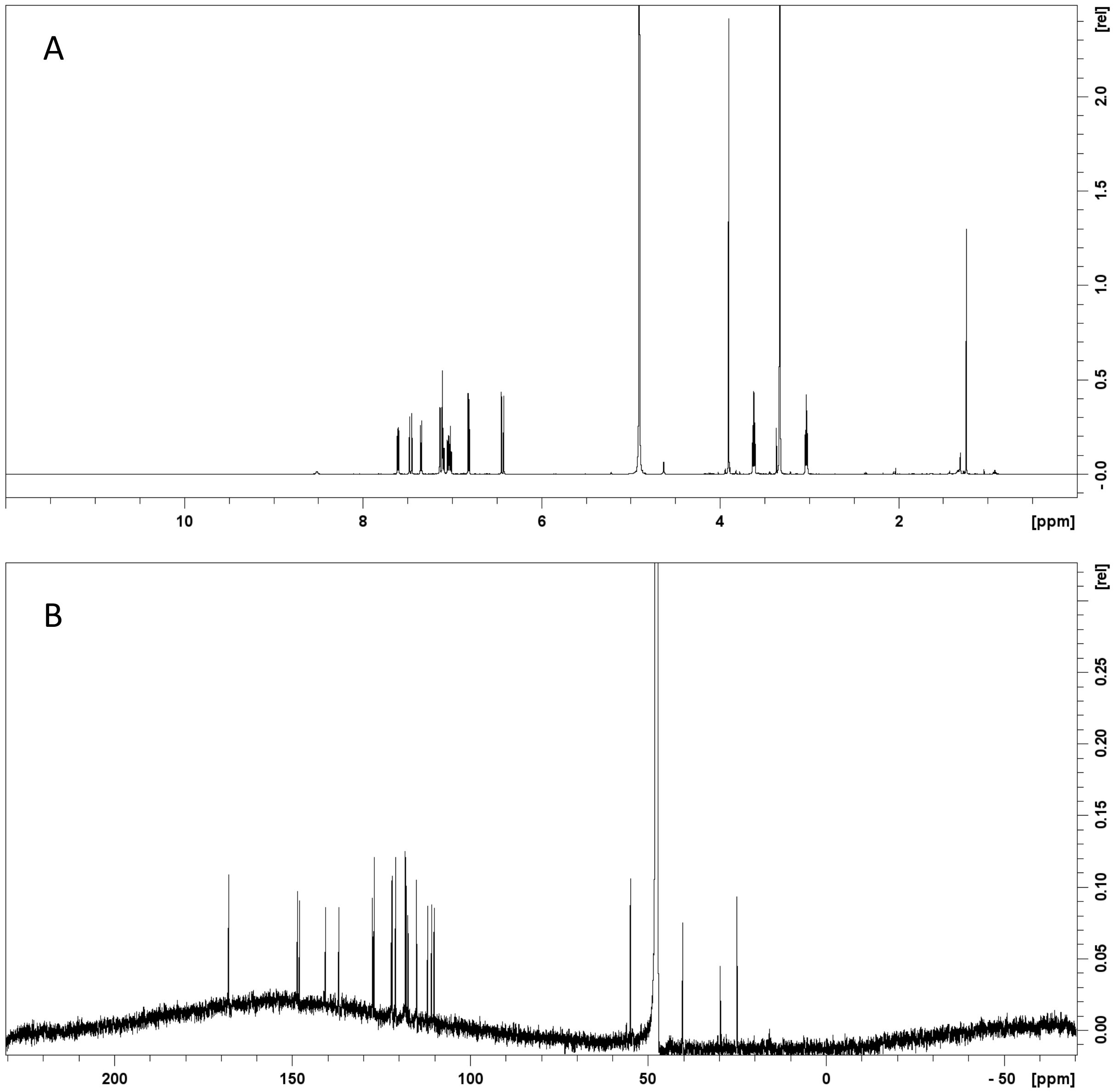

Figure S19 1H (A) and 13C (B) NMR spectra for FerTry

References

[9] Negrel, J.; Smith, T. A., *Phytochemistry* **1984,** *23*, 739-741.

[15] Stöekigt, J.; Zenk, M., *Z. Naturforsch. C* **1975,** *30*, 352-358.

[16] Muroi, A.; Ishihara, A.; Tanaka, C.; Ishizuka, A.; Takabayashi, J.; Miyoshi, H.; Nishioka, T., *Planta* **2009,** *230*, 517.
